# Structured plant metabolomics for the simultaneous exploration of multiple factors

**DOI:** 10.1038/srep37390

**Published:** 2016-11-17

**Authors:** Nikolay Vasilev, Julien Boccard, Gerhard Lang, Ulrike Grömping, Rainer Fischer, Simon Goepfert, Serge Rudaz, Stefan Schillberg

**Affiliations:** 1 Department of Plant Biotechnology, Fraunhofer Institute for Molecular Biology and Applied Ecology IME, Aachen 52074, Germany; 2 School of Pharmaceutical Sciences, University of Lausanne and University of Geneva, 1211, Switzerland; 3 Philip Morris International R&D, Philip Morris Products S.A., Neuchâtel 2000, Switzerland; 4 Department II–Mathematics, Physics and Chemistry, Beuth University of Applied Sciences, Berlin 13353, Germany; 5 Institute for Molecular Biotechnology, RWTH Aachen University, Aachen 52074, Germany

## Abstract

Multiple factors act simultaneously on plants to establish complex interaction networks involving nutrients, elicitors and metabolites. Metabolomics offers a better understanding of complex biological systems, but evaluating the simultaneous impact of different parameters on metabolic pathways that have many components is a challenging task. We therefore developed a novel approach that combines experimental design, untargeted metabolic profiling based on multiple chromatography systems and ionization modes, and multiblock data analysis, facilitating the systematic analysis of metabolic changes in plants caused by different factors acting at the same time. Using this method, target geraniol compounds produced in transgenic tobacco cell cultures were grouped into clusters based on their response to different factors. We hypothesized that our novel approach may provide more robust data for process optimization in plant cell cultures producing any target secondary metabolite, based on the simultaneous exploration of multiple factors rather than varying one factor each time. The suitability of our approach was verified by confirming several previously reported examples of elicitor–metabolite crosstalk. However, unravelling all factor–metabolite networks remains challenging because it requires the identification of all biochemically significant metabolites in the metabolomics dataset.

Secondary metabolites play an important role in the adaptation of plants to environmental stress^1^. Plants react to exogenous factors such as nutrients, hormones and light through signalling pathways that induce downstream stress responses including the modulation of gene expression and the regulation of a broad range of biochemical processes, resulting in the remodelling of metabolism^2^. Key signalling molecules include Ca^2+^, nitrates, phosphates, 2,4-dichlorophenoxyacetic acid (2,4-D), naphthalene acetic acid (NAA), indole acetic acid (IAA), 6-benzylaminapurine (BAP), kinetin, abscisic acid (ABA), jasmonates, salicylic acid, gibberellic acid (GA_3_), ethylene, polyamines, cyclic nucleotides (cAMP and cGMP) and diacylglycerol^2^,^3^,^4^. The accumulation of metabolites in stressed plants may also have economic significance^1^ because signalling components or elicitors can be used to trigger the production of secondary metabolites in whole plants or plant cell and tissue cultures^5^.

Design of experiments (DOE) approaches are used to study the influence of multiple factors simultaneously, allowing the influence of each factor to be determined regardless of other parameters while maintaining independence between the assessment of different effects. This contrasts with the classic ‘one factor at a time’ approach, which is laborious, time consuming and lacks the ability to provide a global picture of molecular events^6^. Factorial designs have recently flourished in plant biology, where they have been used to optimize cultivation parameters for cell and tissue cultures^7^,^8^ and to increase the yield of metabolites^9^,^10^ or recombinant proteins^11^ by medium optimization. However, most of these applications of DOE featured a small number of response variables when describing the corresponding system or process. A much more comprehensive multivariate strategy is needed to identify multiple inducible biomarkers in the plant metabolome following the application of diverse elicitors, so the combination of DOE and metabolomics is an attractive approach for the systematic evaluation of changes in plant secondary metabolism^12^.

Metabolomics generates large, multi-dimensional datasets using automated analytical procedures such as gas chromatography or high-pressure liquid chromatography coupled to mass spectrometry (GC-MS and HPLC-MS). It is therefore necessary to reduce the dimensionality of the data using multivariate statistical methods. The complexity of data mining is enhanced further when the data originate from several sources (e.g. complementary chromatography systems or ionization modes) and data fusion strategies are therefore required. An additional difficulty is encountered when multiple input factors are varied simultaneously, because different sources of variation are mixed. The importance of multiple simultaneous metabolic effects has been underestimated in the past and here we addressed this challenge by combining several orthogonal techniques: reversed-phase ultra-high-pressure liquid chromatography (RP-UHPLC) with positive and negative electrospray ionization (ESI) modes, and hydrophilic interaction liquid chromatography (HILIC), both coupled to time of flight mass spectrometry (TOF-MS) to achieve greater coverage of the metabolome.

Several strategies have been developed for the simultaneous analysis of multiple datasets. The proposed data modelling approach is an extension of the multiple kernel learning method to orthogonal partial least squares discriminant analysis (OPLS-DA), i.e. consensus OPLS-DA, which combines data blocks using the weighted sum of X·X^T^ product association matrices corresponding to their linear kernel^13^. The OPLS-DA framework is advantageous for data interpretation because relevant metabolic variations are associated with predictive components, whereas unrelated variation is summarized in so-called orthogonal components^14^. In consensus OPLD-DA, the block weighting is based on modified RV-coefficients so that the Y response orientates the consensus kernel towards improved predictability. Cross-validation is carried out to assess the optimal model size and avoids overfitting, using DQ^2^ (an adaptation of the conventional Q^2^ value) for discriminant analysis^15^.

To our knowledge, this is the first systematic investigation of metabolic remodelling in plants following simultaneous multi-factorial treatment. This novel combination of metabolomics and experimental design, associated with the simultaneous analysis of multiblock omics data, is a powerful approach that allows us to unravel the metabolic responses in transgenic tobacco cells at a global level when diverse input factors such as macronutrients, plant growth regulators and light are varied simultaneously. Furthermore, this high-throughput screening system can be used for process optimization with metabolically engineered cell lines. Herein we hypothesize that product optimization using the simultaneous exploration of multiple factors may achieve more accurate and reproducible results than the assessment of one factor at a time.

## Results

### UHPLC-QTOF-MS fingerprinting

The acquisition of high-quality metabolomics data is an essential aspect of metabolic profiling because it facilitates the identification step. The UHPLC-QTOF-MS gradient conditions we applied allowed us to monitor more than 3500 features in the *m*/*z* range 100–1200 based on RP-UHPLC (ESI^+^ and ESI^−^ ion detection modes) and HILIC (ESI^+^ ion detection mode). Pareto scaling was applied as data normalization. The multiblock data fusion strategy we used allows the integration of datasets originating from different ionization (ESI^+^/ESI^−^) and separation (RP/HILIC) modes, yielding 1500, 1366 and 1368 variables for the RP/ESI^+^, RP/ESI^−^ and HILIC/ESI^+^ blocks, respectively. Some of the signals were present in all blocks, whereas others were found in only one or two data blocks and thus provided complementary information^13^. The target metabolites in methanol extracts, obtained from tobacco suspensions expressing *Valeriana officinalis* geraniol synthase (*VoGES*) gene, were identified based on mass fragmentation and the comparison of characteristic *m/z* species with internal or publicly accessible natural product databases.

### Consensus OPLS-DA data modelling and metabolite identification

A supervised data mining approach (consensus OPLS-DA) was applied to determine the distinct metabolic changes caused by the simultaneous modulation of diverse input factors, and leave-one-out cross-validation (LOOCV) was carried out to evaluate the appropriate number of orthogonal components based on the DQ^2^ parameter. A series of 1000 permutation tests was performed for each model by randomizing the original Y class response in order to assess the statistical validity of the models^13^.

Significant models were obtained for most of the factors with a prediction accuracy (PA) of 87–100% and a statistical significance of p < 0.01. Lower prediction accuracy was observed for GA_3_ (PA = 62.5%, p = 0.013) and ethephon (PA = 60.4%, p = 0.015), whereas KH_2_PO_4_ (PA = 57.3%, p = 0.225) and MgSO_4_ (PA = 47.9%, p = 0.62) were found to be statistically non-significant (Table 1). Random permutations of the design matrix simulate data under the null hypothesis, i.e. no effect of the experimental factor under study. Because similar or higher prediction accuracies could be achieved from randomly permutated designs, we decided to omit KH_2_PO_4_ and MgSO_4_ from subsequent experiments aiming to identify metabolites and to determine their biological importance.

Next we used S-plots to highlight relevant metabolites based on their contributions to the model, reflecting both their amplitude of variation and their reliability^14^. This visualization method helps to identify biochemically significant metabolites based on their position in the S-plots. The ideal biomarker has a high covariance magnitude and high correlation reliability. This low risk of spurious correlations corresponds to the upper right quadrant (area 1) or lower left quadrant (area 4) of each S-plot. The biomarkers located in area 1 are associated with metabolites that become more abundant when the factor strength is enhanced or less abundant when the factor strength is reduced, i.e. an upregulation effect (metabolic levels^(+)^/factor^(+)^ or metabolic levels^(−)^/factor^(−)^). Similarly, the biomarkers in area 4 are linked to metabolites that become less abundant when the factor strength is enhanced or more abundant when the factor strength is reduced, i.e. a downregulation effect (metabolic levels^(−)^/factor^(+)^ or metabolic levels^(+)^/factor^(−)^). Because all three data blocks (RPLC NEG, RPLC POS and HILIC) are integrated in a single model for each factor, ions detected by each analytical protocol can be associated in a combined S-plot.

High-resolution QTOF-MS/MS profiling allowed 45 compounds represented by some of the most significant features with respect to the factors we investigated (i.e. they were located in areas 1 and 4 of the S-plots) to be tentatively identified, at least to the level of the compound class (Table 2). However, the complete identification of all other relevant metabolites remains challenging and these preliminary results may serve as a starting point for the further targeted isolation and purification of the metabolites of interest.

### Simultaneous treatments reveal nutrient–metabolite and elicitor–metabolite networks

Our primary goal was to identify metabolites that respond to each factor treatment when multiple factors are modulated simultaneously. Using this approach, we avoided the restriction of experimental results caused by the artificially narrow experimental conditions of the one factor at a time approach. In the following sections, bold numbers in parentheses represent the compounds from the list of identified metabolites (Table 2).

### Nutrients

**Figure 1:**
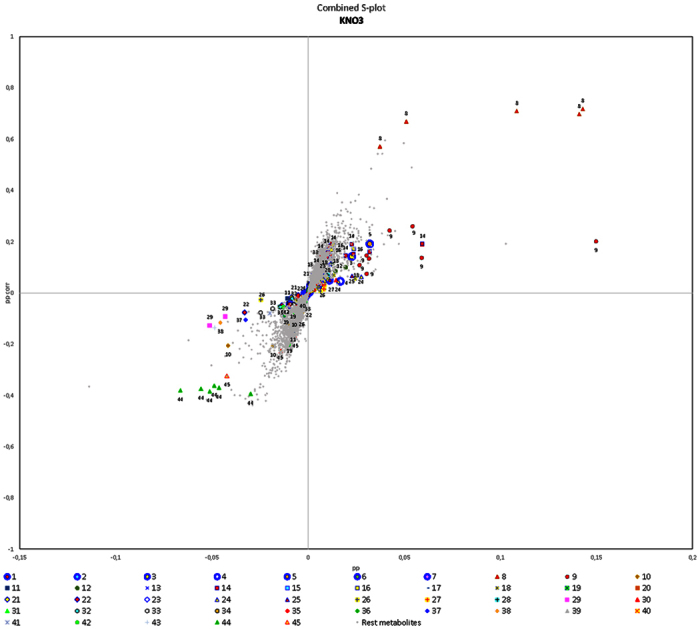
KNO_3_ S-plot showing the distribution of metabolites in transgenic tobacco cell cultures exposed to different combinations of environmental factors, revealing metabolites in areas 1 (upper right) and 4 (lower left) that are the most sensitive to changes in KNO_3_ levels. Numbers refer to compounds listed in Table 2.

KNO_3_ treatment (Fig. 1) increased the abundance of several ions representing glutamine (**8**) and scopolin (**9**), but also single ions representing a 2,4-D metabolite (**14**) (t_R_ = 8.85 min; *m/z* 559.2755) and hexosyl geranidiol (**5**) (t_R_ = 5.96 min; *m/z* 355.1749). In contrast, KNO_3_ caused the extracellular loss of potassium ions, detected as K^+^ (KHCOO)_n_ adducts (**44**), and inhibited the formation of nicotine (**45**), hydroxysphingosine (**10**) and C16:3 monoacylglycerol (**29**). Single ions representing the following compounds were also less abundant following treatment with KNO_3_: trihydroxy-C18:2 acids (**37**, **38**) (t_R_ = 11.36/11.18 min; *m/z* 327.2170/327.2171), a dihydrozeatin (DHZ) adduct (**22**) (t_R_ = 3.26 min; *m/z* 354.1794), a cyclanilide metabolite (**33**) (t_R_ = 9.26 min; *m/z* 620.0896) and BAP glycoside (**26**) (t_R_ = 4.08 min; *m/z* 388.1647).

NH_4_NO_3_ treatment (Fig. S1) induced the synthesis of the monoterpenoid derivatives malonyl-hexosyl-geraniol (**4**), pentosyl-hexosyl-geraniol (**2**) and malonyl-hexosyl-geranidiol (**7**) as well as scopolin (**9**). Two single ion species representing BAP glycosides were also more abundant following treatment with NH_4_NO_3_, i.e. BAP glucoside (**26**) (t_R_ = 3.94 min; *m/z* 388.1634) and BAP riboside (**27**) (t_R_ = 6.59 min; *m/z* 358.1532), as well as a salicylic acid glucoside (**30**) (t_R_ = 3.31 min; *m/z* 323.0759). In contrast, auxin metabolites representing NAA (**18**) and 2,4-D (**14**) as well as single ions representing conjugated dihydroxy-C18:2 fatty acid (**43**) and hexosyl-hexosyl-geraniol (**6**) were less abundant following treatment with NH_4_NO_3_.

The formation of three metabolites was strongly induced by calcium treatment: hydroxysphingosine (**10**), scopolin (**9**) and hexosyl-hexosyl-geraniol (**6**). Adenosine (**11**) synthesis was also stimulated by calcium and was represented by a single ion (t_R_ = 2.65 min; *m/z* 268.1082) (Fig. S2). Several metabolites became less abundant following calcium treatment, namely C16:3 monoacylglycerol (**29**), geraniol derivatives produced by the heterologous geraniol synthase (**1**, **2**, **4** and **7**), and a fatty acid with conjugated triene (**40**) (t_R_ = 12.15 min; *m/z* 325.2013).

### Auxins

Numerous metabolites were influenced by IAA (Fig. S3). We observed an increase in the abundance of indole-3-carboxylic acid glucoside (**17**) and K^+^ (**44**). The monoterpenoid derivatives pentosyl-hexosyl-geraniol (**2**) and malonyl-hexosyl-geraniol (**4**), a DHZ glycoside (**21**) (t_R_ = 6.56 min; *m/z* 384.1906) and its adduct (**22**), C16:3 monoacylglycerol (**29**) and nicotine (**45**) (t_R_ = 3.8096 min; *m/z* 163.1254) were also more abundant following treatment with IAA. The formation of other metabolites was inhibited, particularly adenosine (**11**), scopolin (**9**), BAP glucoside (**26**), and the geraniol derivatives pentosyl-hexosyl-geranidiol (**1**) (t_R_ = 5.76 min; *m/z* 487.2159), hexosyl-geranidiol (**5**) and malonyl-hexosyl-geranidiol (**6**) (t_R_ = 8.96 min; *m/z* 501.2312).

Indole-3-butyric acid (IBA) treatment (Fig. S4) induced the synthesis of IBA glycoside (**20**), geraniol glycosides (**2**, **4**) and C16:3 monoacylglycerol (**29**) but caused the loss of other metabolites including geraniol derivatives (**1**, **5**, **6**).

NAA treatment (Fig. S5) mainly increased the abundance of its own derivatives (**18**, **19**). The following metabolites became less abundant: several single ions characteristic of fatty acids with conjugated triene (**40**–**42**), trihydroxy-C18:2 acid (**37**), indole-3-carboxylic acid glucoside (**17**) (t_R_ = 4.68 min; *m/z* 346.0923), pentosyl-hexosyl-geraniol (**2**), malonyl-hexosyl-geraniol (**4**) and nicotine (**45**).

The formation of several compounds was induced by 2,4-D treatment (Fig. S6): 2,4-D metabolites (**14**–**16**), adenosine (**11**) and the geraniol derivatives (**1**, **5**, **6**). Pentosyl-hexosyl-geraniol (**2**), malonyl-hexosyl-geraniol (**4**) and C16:3 monoacylglycerol (**29**) became less abundant.

Our observations show that naturally occurring (endogenous) auxins such as IAA and IBA upregulate (**2** and **4**) and downregulate (**1**, **5** and **6**) the synthesis of geraniol glycosides, whereas the synthetic auxins (NAA and 2,4-D) have exactly the opposite effects on these metabolites.

### Cytokinins

The presence of kinetin (Fig. S7) strongly induced the synthesis of kinetin glucoside (**24**) and kinetin riboside (**25**), and to a lesser extent the geraniol derivatives pentosyl-hexosyl-geraniol (**2**), malonyl-hexosyl-geraniol (**4**) and malonyl-hexosyl-geranidiol (**7**) as well as trihydroxy-C18:2 acid (**37**). DHZ treatment (Fig. S8) induced the formation of three derivatives: DHZ glycoside (**21**), DHZ adduct (**22**) and DHZ metabolite (**23**). BAP treatment (Fig. S9) resulted in the appearance of three derivatives: BAP glucoside (**26**), BAP riboside (**27**) and BAP ribotide (**28**).

### Other plant growth regulators

Methyljasmonate (MeJa) treatment (Fig. S10) only induced the formation of C16:3 monoacylglycerol (**29**) to a significant extent, but we also observed smaller increases in the abundance of a cyclanilide metabolite (**33**), hydroxysphingosine (**10**), nicotine (**45**), unidentified fatty acids with conjugated triene (**41**, **42**), trihydroxy-C18:2 acids (**37**, **38**), adenosine (**11**) and a 2,4-D metabolite (**14**). The only heterologous monoterpenoids induced by MeJa were pentosyl-hexosyl-geraniol (**2**) and malonyl-hexosyl-geranidiol (**7**). The formation of scopolin (**9**) was strongly inhibited, whereas the synthesis of hexosyl-hexosyl-geraniol (**6**), BAP riboside (**27**) and abscisic acid glycoside (**35**) was more weakly downregulated.

Salicylic acid treatment (Fig. S11) mainly induced the formation of its polar derivatives salicylic acid glucoside (**30**) and salicylic acid dihexosyl-glucoside (**31**), but also C16:3 monoacylglycerol (**29**), trihydroxy-C18:2 acid (**38**) and the unidentified fatty acids with conjugated triene (**41**–**43**). The monoterpenoids pentosyl-hexosyl-geraniol (**2**) and malonyl-hexosyl-geraniol (**4**) were also more abundant. Salicylic acid treatment inhibited the formation of a cyclanilide metabolite (**32**), a 2,4-D metabolite (**14**), scopoletin (**12**), a scopoletin derivative (**13**), hexosyl-hexosyl-geraniol (**6**) and BAP riboside (**27**).

GA_3_ treatment (Fig. S12) induced the formation of C16:3 monoacylglycerol (**29**), pentosyl-hexosyl-geranidiol (**1**), trihydroxy-C18:2 acid (**37**), BAP glucoside (**26**), adenosine (**11**), malonyl-hexosyl-geranidiol (**7**), hexosyl-geranidiol (**5**), NAA metabolites (**18**, **19**) and the unidentified fatty acid with conjugated triene (**42**). It inhibited the formation of scopolin (**9**), a 2,4-D metabolite (**14**), hexosyl-geraniol (**3**), hexosyl-hexosyl-geraniol (**6**), a DHZ adduct (**22**) and a cyclanilide metabolite (**32**).

Ethephon treatment (Fig. S13) induced the formation of C16:3 monoacylglycerol (**29**), pentosyl-hexosyl-geraniol (**2**), BAP glucoside (**26**), trihydroxy-C18:2 acid (**38**), scopolin (**9**) and malonyl-hexosyl-geraniol (**4**), but inhibited the formation of a DHZ adduct (**22**), adenosine (**11**), hydroxysphingosine (**10**), hexosyl-geranidiol (**5**), hexosyl-hexosyl-geraniol (**6**) and a cyclanilide metabolite (**32**).

Cyclanilide treatment (Fig. S14) induced the formation of its own metabolites (**32**–**34**) as well as one NAA metabolite (**18**), hexosyl-hexosyl-geraniol (**6**), unidentified fatty acids with conjugated triene (**40**–**42**), and trihydroxy-C18:2 acids (**37**, **38**). The formation of numerous compounds was downregulated, including pentosyl-hexosyl-geraniol (**2**), malonyl-hexosyl-geraniol (**4**), C16:3 monoacylglycerol (**29**), pentosyl-hexosyl-geranidiol (**1**), conjugated dihydroxy-C18:2 fatty acid (**43**), scopolin (**9**), scopoletin (**12**) and malonyl-hexosyl-geranidiol (**7**).

ABA treatment (Fig. S15) resulted in the accumulation of ABA glycoside (**35**), an ABA metabolite (**36**) and its glycoside (**39**), trihydroxy-C18:2 acids (**37**, **38**), and to a lesser degree the unidentified fatty acids with conjugated triene (**40**–**42**), pentosyl-hexosyl-geranidiol (**1**) and malonyl-hexosyl-geranidiol (**7**). The abundance of DHZ (**22**), a 2,4-D metabolite (**14**), a cyclanilide metabolite (**32**), kinetin riboside (**25**), scopolin (**9**), and DHZ glycoside (**21**) declined in response to ABA.

### Light

Light was the only physical factor we included in our experimental design, and it was associated with an increase in the levels of adenosine (**11**), two unidentified fatty acids with conjugated triene (**41**, **42**), trihydroxy-C18:2 acids (**37**, **38**), malonyl-hexosyl-geranidiol (**7**), and to a lesser extent glutamine (**8**), a 2,4-D metabolite (**14**) and C16:3 monoacylglycerol (**29**). Light inhibited the production of alkaloids, i.e. scopoletin (**12**), a scopoletin derivative (**13**) and scopolin (**9**), and the monoterpenoids pentosyl-hexosyl-geraniol (**2**), hexosyl-geraniol (**3**), and malonyl-hexosyl-geraniol (**4**). A DHZ adduct (**22**) and hydroxysphingosine (**10**) were also less abundant under strong illumination (Fig. S16).

### Clustering

**Figure 2:**
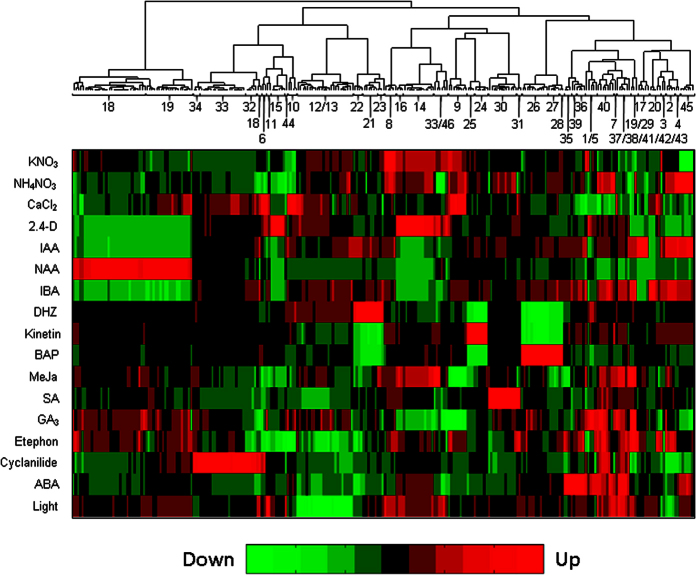
Cluster analysis and heat map.

Cluster analysis provided a global overview of regulation events that follow changes in the experimental factors. This approach allows the grouping of metabolites with similar ion features using a dendrogram, and a heat map summarizes the contribution of each of the factors in the context of each identified metabolite and thus enables the visualization of upregulation and downregulation in response to different treatments (Fig. 2).

Both geranidiol glycosides (**1** and **5**) are located in a small cluster, whereas the third geranidiol derivative (**7**), an esterified monoglycoside, is found in a neighbouring cluster (Fig. 2). The geraniol metabolites (**1** and **5**) are upregulated by 2,4-D, DHZ, GA_3_ and ABA and downregulated by NH_4_NO_3_, CaCl_2_, IAA, IBA, BAP, salicylic acid and ethephon, whereas the upregulation of compound (**7**) is related to NH_4_NO_3_, kinetin, MeJa, GA_3_, ABA, and light, and its downregulation is associated with CaCl_2_, NAA, BAP and cyclanilide. The geraniol glycosides (**2**, **3** and **4**) are located in a common larger cluster. These three metabolites are upregulated by IAA, IBA, kinetin and ethephon, but downregulated by CaCl_2_, 2,4-D, NAA and light. Compounds (**2** and **4**) are distinguished from hexosyl-geraniol (**3**) mainly by the action of NH_4_NO_3_, i.e. (**2** and **4**) are upregulated whereas (**3**) is downregulated by NH_4_NO_3_. The heat map also shows the upregulation of hexosyl-hexosyl-geraniol (**6**) by CaCl_2_, 2,4-D, BAP and cyclanilide, and its downregulation by NH_4_NO_3_, IAA, IBA, DHZ, MeJa, salicylic acid, GA_3_, ethephon and ABA.

## Discussion

The combination of fractional factorial design and consensus OPLS-DA methods allowed us to systematically explore the effect of multiple factors on plant metabolism, using transgenic tobacco cell cultures as a model system. This simultaneous application of treatment stress assesses all experimental factors under diverse conditions that could occur in nature. We tentatively identified 45 constituents in areas 1 and 4 of the S-plots following the fractionation and analysis of plant cell extracts by UHPLC-QTOF-MS, corresponding to metabolites whose abundance changed substantially in response to the experimental factors. These metabolites represented multiple classes of natural products: monoterpenoids, nitrogen-containing compounds, coumarins, fatty acids and their esters, and derivatives of phytohormones and plant growth regulators used as additives in the experiments.

Geraniol and its glycosides do not occur naturally in tobacco plants and their presence in our samples reflects the activity of the stably integrated *VoGES* gene^16^. However, the glycosylation profile of geraniol produced by our cell suspension cultures differed to that observed in whole plants. The cells produced seven distinct geraniol glycosides whereas 19 variants were produced by transgenic tobacco plants and *Nicotiana benthamiana* leaves used for transient expression. The acetylated glycosides produced at later stages of plant development were not monitored in our plant cell cultures. The cell suspension cultures produced geraniol monoglycosides and diglycosides, whereas the whole plants also produced geraniol glycosides with three or more sugar adducts. However, our cell suspension cultures accumulated geranidiol derivatives that are not produced in agroinfiltrated or transgenic plants, which instead produce geranic acid glycosides^16^.

Nitrogenous compounds are found in tobacco cells because they have multiple core metabolic functions and are also precursors in the biosynthesis of tobacco alkaloids^17^. Phytoalexins defend plants against biotic and abiotic stress^18^. The coumarin scopoletin (**12**) is one of the phytoalexins produced in tobacco^19^,^20^,^21^. C16:3 monoacylglycerol (**29**) is a glyceride which can be formed by the esterification of glycerol with one fatty acid or by enzymatic hydrolysis of a fatty acid from diacylglycerol by the action of diacylglycerol lipase. Diacylglycerol acts as a signalling molecule during plant development and in response to stress tolerance, nutrient deficiency and other environmental stimuli^22^,^23^,^24^.

Our metabolomic analysis showed that plant cells react strongly to phytohormones and plant growth regulators. Plants limit the impact of harmful xenobiotic compounds by hydroxylation, glutathione conjugation, glycosylation, malonylation and sulfonylation^25^,^26^. Most of the phytohormone and growth regulator derivatives we identified were polar glucosylated products, which are generally more soluble than the parent molecule thus facilitating elimination. We also detected malonylated geraniol glycosides, confirming that malonylation is one of the key mechanisms used by tobacco cells to metabolize xenobiotic compounds^27^.

The production of glutamine was strongly upregulated by KNO_3_ treatment in our experiment. Nitrate is assimilated by plant cells from nitrite and ammonium, and is then converted into the amino acid glutamine^28^. The protein kinase CIPK23 is involved in both nitrate and potassium signalling^29^. CIPK23 phosphorylates nitrate transporter NPF6.3 after interacting with the calcineurin-B-like protein CBL9, and reduces nitrate uptake capacity in the presence of high external NO_3_^−^ concentrations, whereas the CBL1-CIPK23 and CBL9-CIPK23 complexes activate the K^+^ channel AKT1^30^. A monoacylglycerol derivative (**29**) was also less abundant following KNO_3_ treatment. This may be a hydrolysis product of diacylglycerol which activates protein kinase C and induces nitrate reductase gene expression^31^.

The NO_3_^−^/NH_4_^+^ ratio in the culture medium influences the activity of auxins and cytokinins^32^. We observed an increase in the abundance of ions representing 2,4-D and the loss of ions representing DHZ metabolites and BAP glycoside in the KNO_3_ S-plot, but an increase in the abundance of BAP glycosides and the loss of ions representing 2,4-D and NAA metabolites in the NH_4_NO_3_ S-plot. This indicates that the balance between NO_3_^−^ and NH_4_^+^ ions may affect phytohormone sensitivity.

NH_4_NO_3_ and CaCl_2_ had the most significant impact on the biosynthesis of geraniol glycosides among the inorganic factors we tested. NH_4_NO_3_ induced the formation of geraniol glycosides (**2**, **4**, **7**) but inhibited the formation of hexosyl-hexosyl-geraniol (**6**), whereas calcium showed the opposite behaviour. Higher concentrations of useable nitrogen also enhanced the accumulation of linalool and citronellol by *Saccharomyces cerevisiae*^33^. Geraniol blocks calcium and potassium channels in mammalian cells^34^ and similar cyclic nucleotide-gated ion channels are found in plants^35^.

Ca^2+^ induced the hydroxylation of sphingosine and adenosine agreeing with previous observations that sphingosine-1-phosphate increases cytosolic free Ca^2+^ 36 and cyclic adenosine monophosphate regulates calcium channels in the plasma membrane of *Arabidopsis thaliana* leaf guard and mesophyll cells^37^.

Scopolin and its 7-O-glucoside are key components of the abiotic stress response^18^ and the abundance of both compounds increased following treatment with all three statistically significant nutrients in our study. The accumulation of scopoletin in tobacco cells and its conversion to a glucoside is also induced by 2,4-D^21^. Ions representing scopolin were also detected following the treatment of our cells with 2,4-D. Scopoletin synthesis was strongly inhibited by MeJa concurring with data showing that scopoletin biosynthesis induced by *Alternaria alternata* is strongly dependent on jasmonic acid but not ABA, although MeJA does not induce scopoletin production in the absence of *A. alternata*^38^. In our cells, the formation of scopoletin was also inhibited by GA_3_.

We detected nicotine produced in trace amounts by our transgenic tobacco cell cultures, which comprise green (photosynthesizing) cells derived from the aerial parts of the plant, although *de novo* nicotine synthesis takes place mainly in the roots^39^. MeJa induced nicotine production in our tobacco cells in a similar manner as previously shown for *N. attenuata*^39^. The downregulation of nicotine production we observed following the treatment with KNO_3_ and NAA agrees with previous reports for cultured tobacco callus, and the effect of K^+^ is probably mediated by NAA^40^. We also observed that nicotine biosynthesis was induced by IAA but moderately suppressed by 2,4-D^41^. The combined effect of MeJa, auxins and K^+^ on the regulation of nicotine synthesis suggests that multiple factors contribute to the same process.

The biosynthesis of monoterpenoid glycosides appeared to be influenced by auxins and cytokinins, perhaps reflecting the antagonistic crosstalk between these two phytohormone classes^42^. Our experimental results support the idea that phytohormones function in a complex network involving the different hormonal pathways but that there is also elaborate crosstalk with nutrients and elicitors. The auxin-sensitive signalling protein SHY2 is regulated by the cytokinin-induced protein ARR1 (*Arabidopsis* response regulator), which in turn is repressed by gibberellin thus connecting three hormones in one network^42^. This may explain why our GA_3_ S-plot contained ion traits that were also affected by auxins and cytokinins. We also observed evidence for ethylene/cytokinin and cytokinin/ABA crosstalk^42^.

Light has a potent effect on monoterpenoid metabolism by modulating the expression of monoterpenoid synthase genes, controlling precursor synthesis, and affecting constitutive promoter activity^43^,^44^. Geraniol glycosides were also influenced by light in our cell cultures. Light induced the formation of malonyl-hexosyl-geranidiol (**7**) but suppressed the formation of pentosyl-hexosyl-geraniol (**2**), hexosyl-geraniol (**3**) and malonyl-hexosyl-geraniol (**4**).

We have developed a systematic approach, which implements an experimental design strategy in the context of metabolomics to account for the diverse factors applied simultaneously to plant cells. This is a valuable method for the investigation of complex environmental stress and its impact on plant metabolism by optimizing the number of experiments needed to assess the factors. Our approach significantly reduces the time and effort required for testing by using consensus OPLS-DA models to evaluate and interpret metabolic changes caused by the simultaneous application of diverse ecological factors. This systematic workflow may facilitate the discovery and characterization of factor–nutrient–elicitor networks and appropriate biomarkers. Finally, we conclude that this novel approach should be able to streamline process optimization for the reproducible production of any secondary metabolite in plant cell cultures by the simultaneous exploration of multiple factors rather than the assessment of one factor at a time.

## Materials and Methods

### Plant cell cultures, treatments and harvesting

We used tobacco (*N. tabacum* cv. Samsun NN) transgenic cell suspension cultures, expressing stably *V. officinalis* geraniol synthase. The cell cultures were initiated and maintained as previously described^8^. Two levels (low/high) were used for each of the factors selected for analysis. The low level of each macronutrient in the plant cell culture medium was based on classical Murashige and Skoog (MS) medium^11^,^45^ whereas the high level was based on our recent medium optimization study, although the concentrations of NH_4_NO_3_ were reversed^11^,^45^. The specific low/high concentrations were prepared as follows: KNO_3_ (18.79/70.16 mM), CaCl_2_ (2.99/10.84 mM), KH_2_PO_4_ (1.25/2.72 mM), MgSO_4_ (1.5/3.0 mM) and NH_4_NO_3_ (4.24/20.61 mM). For the auxins, cytokinins and plant regulators (IAA, IBA, NAA, 2,4-D, kinetin, DHZ, BAP, MeJa, salicylic acid, GA_3_, ethephon, cyclanilide and ABA) the low and high levels were set to 0 and 10 μM, respectively. Finally, the low and high levels of light were set to 11.50 and 35.62 μmol/cm^2^/s, respectively^8^. The factor levels are summarized in Table S1.

The following cultivation conditions were used: flask volume 50 ml (Erlenmeyer glass flasks), filling volume 25 ml, inoculum size 1.4 g fresh weight (FW), triacetyl-β-cyclodextrin concentration 2 mM, and shaking frequency 180 rpm^8^. The plant cell suspension cultures were grown for 9 days before the cells were harvested, then filtered twice under vacuum and frozen at −20 °C. The cultures were elicited with phytohormones and plant growth regulators 6 days post-inoculation. The experiment was conducted at 26 °C with a 16-h photoperiod in an ISF1-X shaker (Kühner AG, Birsfelden, Switzerland).

### Sample preparation

The samples were extracted as previously described^46^. Briefly, ~100-mg aliquots of plant material were mixed with methanol (1:3 w/v) in a shaker mill (TissueLyser, Retsch, Haan, Germany) and pulverized with a steel ball at 25 beats per second for 1 min. Homogenized samples were sonicated (15 min), centrifuged (1750 × *g*, 10 min, 25 °C) and supernatants were passed through a 0.2-μm membrane filter.

### UHPLC-MS

The samples prepared above were analysed on an Acquity UPLC system coupled to a QTOF Premier MS detector (Waters, Milford, MA, USA). For RP-UHPLC, a Waters Acquity BEH C18 column (2.1 × 150 mm, 1.7 μm) was used with water containing 0.1% (v/v) formic acid (A) and acetonitrile containing 0.1% (v/v) formic acid (B) as eluents applied as the following gradient: 0 min, 5% B; 23.5 min, 80% B; 24 min, 96% B; 26 min, 96% B. The flow rate was 0.4 ml/min with the column temperature set to 45 °C. HILIC separation was carried out using a Waters Acquity HILIC column (2.1 × 150 mm, 1.7 μm) with 33 mM aqueous ammonium formate, pH 4.5 (A) and acetonitrile (B) as eluents applied as the following gradient: 0 min, 4% A; 3 min, 4% A; 17.5 min, 32% A; 18 min, 55% A; 20 min, 55% A. The flow rate was 0.5 ml/min with the column temperature set to 50 °C.

A pool was created by adding equal volumes from all samples to serve as a QC injection. Nine QC injections in total were distributed at regular intervals in the analytical batch. An acceptable variation was achieved for all peaks, including those with the highest intensity (coefficient of variation less than 40%).

Positive and negative ESI was applied in separate analytical runs (positive only for the HILIC method) with a desolvation gas flow of 780 l/h at 400 °C, a capillary voltage of 4.5 kV and a cone voltage of 45 V. Mass spectra were acquired over the *m/z* range 100–1200 in “W mode” using leucine enkephalin as a lock mass standard.

### Raw data processing

Mass/retention time markers were extracted from the raw UHPLC-ESI-MS data using MarkerLynx XS v4.1 (Waters). The following method parameters were set: retention time window 1.6–24.6 min, *m/z* range 100–1200, XIC window 0.02 Da, peak smoothing activated, marker intensity threshold 30, mass window 0.04 Da, retention time window 0.2 min, noise elimination level 6.0, and deisotope data activated. All mass (*m/z*)/retention-time features related to the auxins, cytokinins and plant growth regulators provided as supplements in the plant cell culture media, as well as features derived from impurities in the LC eluents, were removed from the raw metabolomics datasets by removing all features detected in blank runs (solvent injection) or in analytical runs of the pure additives before data evaluation.

### Experimental design

The experimental design was based on an orthogonal array with 96 runs created with the free open-source R package DoE.base^47^ as described in the supplementary material. Given the size of this experiment, tests for effects of 2-level factors at significance level 5% can detect effects of size “one standard deviation” with about 99% power, effects of size “half a standard deviation” with about 68% power, and effects of size “0.75 standard deviations” with about 95% power. The design was optimized to screen 14 factors by keeping the confounding of low-order effects minimal: all main effects are orthogonal to each other (orthogonal array), the design was based on an array with the lowest possible number of squared canonical correlations from three-factor sets equal to 1^48^ and the factors were accommodated on columns of the base array such that confounding between main effects and two-factor interactions, and subsequently among two-factor interactions, was minimized^8^.

This fractional factorial design, with a randomized run order, was used to screen 12 two-level factors, one three-level factor and one four-level factor: we thus screened for the effects of light and 18 diverse substances representing macronutrients, auxins, cytokinins and elicitors. For the two-level factors, we investigated the presence of the high levels of NH_4_NO_3_, KNO_3_, CaCl_2_, KH_2_PO_4_, MgSO_4_, MeJa, salicylic acid, GA_3,_ ethephon, cyclanilide, ABA and light. For auxins (four-level factor), exactly one of IAA, IBA, NAA or 2,4-D was present, whereas for cytokinins (three-level factor), exactly one of kinetin, DHZ or BAP was present. The experimental design with 96 runs is summarized in coded values in Table S2. The remaining potentially relevant confounding between main effects and two-factor interactions in terms of triples of factor comparisons are summarized in Fig. S17. For each such triple, the comparison between the levels of each factor in the triple might be affected by an interaction between the other two factors (e.g. an interaction between KNO_3_ and NH_4_NO_3_ might affect the assessment of the BAP vs. DH-z comparison for cytokinins). Sceptics might argue that this possibility for confounding is a reason to refrain from using an experimental design approach in favour of only changing one factor at a time (OFAT approach). However, if two-factor interactions are indeed relevant – as would be necessary for the experimental design approach to suffer from misleading conclusions in terms of factor level comparisons – the conclusions from an OFAT approach are also limited in the same manner and would be valid only for the exact settings at which the other factors have been fixed. Furthermore, to achieve a reasonable amount of replication, the OFAT approach would need a much larger number of experimental runs – e.g. 24 runs for the reference level combination (the number obtained for the four-level factor in the 96-run experiment) might be combined with 24 runs each for the other level of the 12 two-level factors (12*24), and 24 runs each for the other levels of the three-level and the four-level factors (5*24), resulting in a total of 18*24 = 432 runs instead of the 96 runs in our experiment.

### Data processing and analysis

For each experimental factor, a consensus OPLS-DA model was built to relate the experimental metabolomics data (X) to a class matrix consisting of zeros and ones, filled according to the levels of each factor (Y). The columns of the experimental design were therefore used individually as a response matrix in the context of supervised analysis. For auxins and cytokinins, a response vector was generated individually for each hormone and filled with zero when the corresponding hormone was absent, whereas a value of one indicated its presence. The consensus OPLS algorithm implements data fusion based on multiple kernel learning. The joint analysis of multiple data tables is achieved by the combination of association matrices computed for each block. Therefore, requirements in terms of memory resources and computation time are minimized without information loss even if the experimental data include a large number of signals. A block-scaling step ensures fairness between blocks by offering equal starting chances to contribute to the model. RV coefficients are then computed to build a consensus matrix and orientate the model towards better prediction performance. A common subspace is built using a kernel version of the OPLS algorithm and the optimal number of orthogonal components is estimated by cross-validation. Because systematic variations are summarized using Y-predictive and Y-orthogonal components (OPLS framework), the interpretation of the multiblock model is straightforward. Like classical multivariate methods, a consensus score plot allows the distribution of the observations to be evaluated. Because linearity is maintained, variable loadings can easily be computed for biomarker discovery. The weight of each block in the projection also allows the role of each data source to be evaluated^13^.

The OPLS model can be summarized as follows:


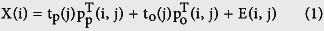



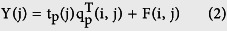


where X contains the normalized metabolomic data from data block i (i ∈ [RPPOS, RPNEG, HILICPOS]), Y represents a 0/1 indicator variable for experimental condition j (j ∈ [NH_4_NO_3_, KNO_3_, CaCl_2_, KH_2_PO_4_, MgSO_4_, IAA, IBA, NAA, 2,4-D, kinetin, DHZ, BAP, MeJa, salicylic acid, GA_3_, ethephon, cyclanilide, ABA, light]), t_p_ is the Y-predictive score matrix, p_p_ is the Y-predictive loading matrix for X, t_o_ is the Y-orthogonal score matrix, p_o_ is the Y-orthogonal loading matrix for X, q_p_ is the Y-predictive loading matrix for Y, and E and F are the residual matrices for X and Y, respectively. Note that the four indicator variables for the cytokinins sum to a column of “+1” entries, as do the three indicator variables for the auxins. Consensus OPLS-DA modelling was carried out under the MATLAB^®^ v8 environment (The MathWorks, Natick, USA) with combinations of toolboxes and in-house functions, including the publicly available RV-coefficients MATLAB m-file^49^ and the KOPLS open source package^50^.

### Cluster analysis

Subsets of metabolites sharing similar patterns were investigated using cluster analysis. For that purpose, the contribution (loading) of each ion feature associated with an identified metabolite was collected across all significant consensus OPLS-DA models and displayed in a dendrogram and a heat map. This strategy highlights upregulation and downregulation. Cluster analysis was carried out with the Bioinformatics Toolbox v4.2 under the MATLAB^®^ v8 environment (The MathWorks) using Euclidean distances and the Ward aggregation method.

### Factors with more than two levels

Auxins and cytokinins were two factors in our design associated with three and four levels, respectively. Exactly one auxin and one cytokinin were included in each run of the experimental design. Consequently, the four Y(j) indicator columns corresponding to auxins and the three Y(j) indicator columns corresponding to cytokinins are linearly dependent, as stated above. Our analysis included all indicator columns, because each is treated separately. This implies that downregulation or upregulation must be interpreted within the linearly-dependent groups, e.g. if the three auxins IAA, IBA and 2,4-D are identified as downregulators, the fourth (NAA) must be an upregulator (relative to the other auxins). This behaviour is clearly shown in the heat map and also implies analogous dependencies among the S-plots of the auxins and cytokinins, respectively.

## Additional Information

**How to cite this article**: Vasilev, N. *et al.* Structured plant metabolomics for the simultaneous exploration of multiple factors. *Sci. Rep.* **6**, 37390; doi: 10.1038/srep37390 (2016).

**Publisher’s note:** Springer Nature remains neutral with regard to jurisdictional claims in published maps and institutional affiliations.

## Supplementary Material

Supplementary Information

## Figures and Tables

**Table 1:** Performance indices of the consensus OPLS-DA models evaluated for each of the screened factors.

	Prediction accuracy	Estimated p-value
KNO_3_	86.5%	<0.001
NH_4_NO_3_	100.0%	<0.001
CaCl_2_	76.0%	<0.001
KH_2_PO_4_	57.3%	0.225
MgSO_4_	47.9%	0.62
2,4-D	97.9%	<0.001
IAA	90.6%	<0.001
NAA	100.0%	<0.001
IBA	99.0%	<0.001
DHZ	100.0%	<0.001
Kinetin	100.0%	<0.001
BAP	100.0%	<0.001
MeJa	99.0%	<0.001
Salicylic acid	96.9%	<0.001
GA_3_	62.5%	0.013
Ethephon	60.4%	0.015
Cyclanilide	97.9%	<0.001
ABA	93.8%	<0.001
Light	97.9%	<0.001

Prediction accuracy was evaluated by leave-one-out-cross-validation, whereas a series of 1000 permutation tests allowed an empirical p-value to be estimated.

**Table 2:** List of identified metabolites.

No.	Metabolite name	Characteristic *m/z*	t_R_(min)	Detection mode
**1**	**pentosyl-hexosyl-geranidiol**	463.2174	5.7656	RPNEG
		487.2159	5.7593	RPPOS
		503.1882	5.7588	RPPOS
**2**	**pentosyl-hexosyl-geraniol**	447.2226	9.5952	RPPOS
		471.2209	9.5930	RPPOS
		487.1962	9.5926	RPPOS
		919.4505	9.6056	RPNEG
**3**	**hexosyl-geraniol**	339.1804	10.3455	RPPOS
		355.1536	10.3461	RPPOS
**4**	**malonyl-hexosyl-geraniol**	425.1792	11.2494	RPPOS
		441.1559	11.2498	RPPOS
**5**	**hexosyl-geranidiol**	355.1749	5.9561	RPPOS
**6**	**hexosyl-hexosyl-geraniol**	477.2335	8.9770	RPNEG
		501.2312	8.9609	RPPOS
**7**	**malonyl-hexosyl-geranidiol**	441.1744	6.9855	RPPOS
**8**	**glutamine**	130.0498	10.6332	HILIC
		147.0752	10.6115	HILIC
		293.1505	10.6280	HILIC
		439.2178	10.6269	HILIC
		585.2814	10.6283	HILIC
**9**	**scopolin**	191.0343	3.5987	RPNEG
		193.0516	3.5994	RPPOS
		193.0535	2.9972	HILIC
		355.1048	3.5976	RPPOS
		355.1061	3.0112	HILIC
		377.0863	3.5995	RPPOS
		399.0922	3.5982	RPNEG
		731.1788	3.5973	RPPOS
**10**	**hydroxysphingosine**	280.266	15.0331	RPPOS
		298.2763	15.0274	RPPOS
		298.2784	5.9910	HILIC
		309.2069	15.0493	RPNEG
		316.2867	15.0264	RPPOS
		316.2884	5.9926	HILIC
		338.2696	15.0462	RPPOS
		382.2419	15.0422	RPPOS
**11**	**adenosine**	136.0569	2.5932	HILIC
		268.1082	2.6470	HILIC
		535.2012	2.6454	HILIC
**12**	**scopoletin**	176.008	5.5960	RPNEG
		178.0277	5.5921	RPPOS
		191.0344	5.5952	RPNEG
		193.0452	5.5936	RPPOS
**13**	**scopoletin derivative**	104.0255	5.5948	RPNEG
		120.021	5.5951	RPNEG
		133.0287	5.5925	RPPOS
		137.0604	5.5922	RPPOS
		148.0156	5.5950	RPNEG
		165.0563	5.5930	RPPOS
		215.0347	5.5938	RPPOS
		292.9786	5.5956	RPPOS
		300.0546	5.5893	RPPOS
		308.0433	5.5902	RPPOS
		316.0304	5.5889	RPPOS
		324.0529	5.5906	RPPOS
		383.0782	5.5973	RPPOS
		405.0578	5.5928	RPNEG
		405.06	5.6036	RPPOS
		423.9898	5.5906	RPPOS
		439.0089	5.5932	RPNEG
		439.0133	5.5951	RPPOS
		629.0385	5.5924	RPNEG
		820.0741	5.5947	RPNEG
**14**	**metabolite of 2,4-D (1)**	160.9559	8.8354	RPNEG
		164.9515	8.8336	RPNEG
		170.0464	8.8283	RPPOS
		174.9733	8.8281	RPPOS
		185.0569	8.8349	RPNEG
		347.0196	8.8379	RPNEG
		349.0374	8.8264	RPPOS
		351.0158	8.8342	RPNEG
		375.0145	8.8278	RPPOS
		415.0057	8.8394	RPNEG
		559.2755	8.8471	RPNEG
		583.2731	8.8317	RPPOS
		717.0307	8.8341	RPNEG
		719.0288	8.8327	RPNEG
		907.3045	8.8428	RPNEG
**15**	**metabolite of 2,4-D (2)**	124.9799	11.6718	RPNEG
		163.9575	11.6759	RPNEG
		164.951	11.6753	RPNEG
		402.9077	11.6770	RPNEG
		404.9042	11.6725	RPNEG
		460.9137	11.6742	RPNEG
**16**	**metabolite of 2,4-D (3)**	170.0482	7.3887	HILIC
		174.9755	7.3879	HILIC
		349.0398	7.3867	HILIC
**17**	**indole-3-carboxylic acid glucoside**	118.0649	4.6888	RPPOS
		262.0714	4.6832	RPNEG
		322.0927	4.6813	RPNEG
		346.0923	4.6828	RPPOS
		669.1911	4.6807	RPPOS
**18**	**metabolite of NAA (1)**	105.0175	5.8475	HILIC
		108.0443	5.8300	HILIC
		109.0283	5.8394	HILIC
		117.0192	5.8314	HILIC
		127.0403	5.8528	HILIC
		141.0702	9.1835	RPNEG
		141.0725	5.8253	HILIC
		145.0526	5.8169	HILIC
		159.0322	5.8371	HILIC
		167.0494	9.1845	RPNEG
		181.0659	9.1840	RPNEG
		199.0765	9.1833	RPNEG
		204.107	5.8286	HILIC
		209.06	9.1827	RPNEG
		227.0711	9.1826	RPNEG
		230.0709	5.8179	HILIC
		231.0554	5.8478	HILIC
		248.0818	5.8441	HILIC
		249.0667	5.8482	HILIC
		266.0926	5.8349	HILIC
		271.0456	9.1705	RPPOS
		339.1097	9.1620	RPPOS
		413.1212	9.1701	RPPOS
		452.1573	5.8402	HILIC
		457.1119	9.1727	RPPOS
		457.1131	5.8504	HILIC
		473.0864	5.8473	HILIC
		473.0876	9.1693	RPPOS
		480.188	5.8748	HILIC
		794.2701	5.8362	HILIC
		815.3345	9.1788	RPNEG
		867.2371	9.1825	RPNEG
		886.2783	5.8310	HILIC
		889.2184	9.1827	RPNEG
**19**	**metabolite of NAA (2)**	101.023	7.9271	RPNEG
		131.0338	7.9234	RPNEG
		132.0298	7.9449	RPNEG
		141.0704	7.9282	RPNEG
		141.0706	7.9397	RPPOS
		161.0452	2.1990	RPNEG
		185.0612	7.9236	RPNEG
		209.0611	7.9270	RPNEG
		221.0668	7.9282	RPNEG
		263.0772	7.9266	RPNEG
		300.0866	7.9416	RPNEG
		324.0873	7.9409	RPPOS
		389.1215	7.9262	RPNEG
		551.1765	7.9259	RPNEG
		614.2078	7.9002	HILIC
		619.1631	7.9120	RPPOS
		623.1633	7.9484	RPNEG
		635.1373	7.9012	HILIC
		918.244	7.9416	RPNEG
		1191.3449	7.9262	RPNEG
**20**	**IBA glycoside**	364.1395	6.4291	RPNEG
		386.1211	6.4297	RPNEG
		388.1387	6.4199	RPPOS
**21**	**glycoside of DHZ**	222.138	6.5705	HILIC
		382.1723	2.2985	RPNEG
		384.1896	2.2933	RPPOS
		384.1906	6.1062	HILIC
		384.1906	6.5631	HILIC
		422.1449	6.5183	HILIC
**22**	**DHZ adduct**	220.1198	3.2585	RPNEG
		222.1346	3.2604	RPPOS
		354.1794	3.2625	RPPOS
		354.1807	2.3623	HILIC
		398.1679	3.2561	RPNEG
**23**	**DHZ metabolite**	148.0651	3.7100	HILIC
		222.1377	3.7152	HILIC
**24**	**kinetin glucoside**	214.0732	2.7567	RPNEG
		216.09	2.7568	RPPOS
		216.0905	4.0422	HILIC
		376.1252	2.7571	RPNEG
		378.1424	2.7578	RPPOS
		378.1442	4.0310	HILIC
**25**	**kinetin riboside**	216.0891	5.0179	RPPOS
		346.1149	5.0193	RPNEG
		348.1326	5.0207	RPPOS
**26**	**BAP glucoside**	224.0928	3.9369	RPNEG
		226.107	4.0927	HILIC
		226.1073	3.9362	RPPOS
		386.1458	3.9367	RPNEG
		388.1634	3.9383	RPPOS
		388.1647	4.0897	HILIC
		410.1452	3.9378	RPPOS
		454.1317	3.9345	RPNEG
		631.2459	4.0379	HILIC
		773.3008	3.9366	RPNEG
		775.3158	4.0637	HILIC
**27**	**BAP riboside**	224.0935	6.5916	RPNEG
		226.1085	6.5889	RPPOS
		356.1355	6.5914	RPNEG
		358.1532	6.5893	RPPOS
		402.1407	6.5909	RPNEG
**28**	**BAP ribotide**	438.1187	4.1593	RPPOS
		598.1542	4.1559	RPNEG
**29**	**C16:3 monoacylglycerol**	325.2295	9.2430	RPPOS
		325.2318	7.0100	HILIC
**30**	**salicylic acid glucoside**	121.0281	3.3077	RPPOS
		137.0229	3.3048	RPNEG
		185.0448	3.3072	RPPOS
		257.1397	3.3079	RPPOS
		299.0764	3.3034	RPNEG
		301.0316	3.3041	RPPOS
		323.0759	3.3067	RPPOS
		599.1603	3.3049	RPNEG
		621.1426	3.3023	RPNEG
		637.1167	3.3054	RPNEG
		653.0792	3.3044	RPNEG
		943.2115	3.3038	RPNEG
**31**	**salicylic acid, dihexosyl-glucoside**	137.0238	3.7150	RPNEG
		485.1274	3.7172	RPPOS
**32**	**cyclanilide metabolite (1)**	177.0566	9.3597	RPPOS
		321.0997	9.3597	RPPOS
		339.1095	9.3596	RPPOS
		934.1961	9.3783	RPNEG
		958.1897	9.3600	RPPOS
**33**	**cyclanilide metabolite (2)**	153.0191	9.2740	RPNEG
		159.973	9.2749	RPNEG
		161.9896	9.2591	RPPOS
		179.056	9.2728	RPNEG
		187.9678	9.2742	RPNEG
		213.0404	9.2755	RPNEG
		227.9976	9.2761	RPNEG
		255.9955	9.2588	RPPOS
		263.0773	9.2750	RPNEG
		271.9874	9.2746	RPNEG
		275.9838	9.2753	RPNEG
		313.9993	9.2750	RPNEG
		347.0969	9.2584	RPPOS
		357.0818	9.2749	RPNEG
		476.0503	9.2743	RPNEG
		498.0332	9.2736	RPNEG
		527.2492	9.4612	RPNEG
		596.0928	9.2734	RPNEG
		618.0751	9.2752	RPNEG
		620.0896	9.2595	RPPOS
**34**	**cyclanilide metabolite (3)**	159.9712	14.1734	RPNEG
		566.9654	14.1686	RPNEG
		568.9623	14.1689	RPNEG
**35**	**ABA glycoside**	153.0915	6.2662	RPNEG
		425.1808	6.2678	RPNEG
		449.1792	6.2491	RPPOS
**36**	**ABA metabolite**	139.076	4.4449	RPNEG
		279.1236	4.4451	RPNEG
		303.1176	4.4479	RPNEG
		465.1742	4.4411	RPPOS
**37**	**trihydroxy-C18:2 acid (1)**	327.217	11.3581	RPNEG
**38**	**trihydroxy-C18:2 acid (2)**	327.2171	11.1797	RPNEG
**39**	**glycoside of abscisic acid metabolite**	281.1393	3.1053	RPNEG
		467.1902	3.0957	RPPOS
**40**	**unidentified fatty acid with conjugatedtriene (1)**	325.2013	12.1543	RPNEG
**41**	**unidentified fatty acid with conjugated triene (2)**	325.2012	10.1746	RPNEG
**42**	**unidentified fatty acid with conjugated triene (3)**	325.2011	9.9604	RPNEG
**43**	**dihydroxy-C18:2 fatty acid (conjugated)**	311.222	13.3326	RPNEG
**44**	**potassium ion**	122.9253	8.4619	HILIC
		206.8899	8.4611	HILIC
		290.8524	8.4597	HILIC
		458.7723	8.4606	HILIC
		542.7322	8.4597	HILIC
		878.5772	8.4627	HILIC
**45**	**nicotine**	106.0650	3.7917	HILIC
		120.0816	3.8033	HILIC
		163.1254	3.8096	HILIC
