# Supplementary material for: Structured plant metabolomics for the simultaneous exploration of multiple factors
Source: Sci Rep. 2016 Nov 17;6:37390. doi: 10.1038/srep37390 (PMC5112604; doi:10.1038/srep37390)

SUPPLEMENTARY INFORMATION

Structured plant metabolomics for the simultaneous exploration of multiple factors

Nikolay Vasilev[1*], Julien Boccard[2], Gerhard Lang[3], Ulrike Grömping[4], Rainer Fischer[1,5], Simon Goepfert[3], Serge Rudaz[2] and Stefan Schillberg[1]

[1]Department of Plant Biotechnology, Fraunhofer Institute for Molecular Biology and Applied Ecology IME, Aachen 52074, Germany

[2]School of Pharmaceutical Sciences, University of Lausanne and University of Geneva, Geneva 1211, Switzerland

[3]Philip Morris International R&D, Philip Morris Products S.A., Neuchâtel 2000, Switzerland.

[4]Department II–Mathematics, Physics and Chemistry, Beuth University of Applied Sciences, Berlin 13353, Germany

[5]Institute for Molecular Biotechnology, RWTH Aachen University, Aachen 52074, Germany

Correspondence and requests for materials should be addressed to N.V. (e-mail: nikolay.vasilev@ime.fraunhofer.de)

R code for design generation

```
require(DoE.base)

show.oas(factors = list(nlevels=c(2,3,4), number=c(12,1,1)), show = "all", showmetrics=TRUE)

Experimental.plan<- oa.design(ID=L96.2.19.3.1.4.23,

nlevels=c(2,2,2,2,2,4,3,2,2,2,2,2,2,2), columns="min34",

        factor.names=list(

        KNO3=c("-","+"), NH4NO3=c("-","+"), CaCl2=c("-","+"), KH2PO4=c("-","+"),

                MgSO4=c("-","+"),

                        auxins=c("IAA", "IBA", "NAA", "2.4-D"),

                                cytokinins=c("kinetin", "DH-zeatin", "BAP"),

                                        MeJa=c("MeJa-","MeJa+"),

                                                SA=c("-","+"),

                                                GA3=c("-","+"),

                                                        Ethephon=c("-","+"), Cyclanilide=c("-","+"),

                                                                AbscA=c("-","+"),

                                                                        Light=c("Lght-","Lght+")),

        seed = 49, randomize=TRUE)

Experimental.plan
```

Table S1. Factor levels.

	**Low level (-)**	**High level (+)**
Nutrients		
**NH_4NO_3**	4.24 mM	20.61 mM
**KNO_3**	18.79 mM	70.16 mM
**$CaCl_2$**	2.99 mM	10.84 mM
**KH_2PO_4**	1.25 mM	2.72 mM
**$MgSO_4$**	1.5 mM	3.0 mM
Auxins		
(10 µM of chosen one; reference level underlined)		
	IAA <u>IBA</u>	NAA 2,4-D
Cytokinins		
(10 µM of chosen one; reference level underlined)		
	Kinetin DHZ	<u>BAP</u>
Elicitors		
**MeJa**	0 µM	10 µM
**Salicylic acid**	0 µM	10 µM
**GA_3**	0 µM	10 µM
**Ethephon**	0 µM	10 µM
**Cyclanilide**	0 µM	10 µM
**Abscisic acid**	0 µM	10 µM
Light	11.5 µmol/s	35.62 µmol/s

Table S2. Experimental design in coded values.

Run name	KNO_3	NH_4NO_3	$CaCl_2$	KH_2PO_4	$MgSO_4$	Auxins	Cytokinins	MeJa	SA	GA_3	Ethephon	Cyclanilide	ABA	Light
1	-	+	-	+	+	IAA	DHZ	MeJa+	+	+	-	+	+	Lght+
2	-	+	+	+	+	NAA	Kinetin	MeJa+	-	+	+	+	+	Lght+
3	-	+	-	-	-	2,4-D	Kinetin	MeJa-	+	+	+	+	+	Lght+
4	+	-	-	+	-	IAA	Kinetin	MeJa+	-	+	-	-	+	Lght+
5	-	-	-	-	+	IAA	DHZ	MeJa-	+	+	+	-	+	Lght+
6	-	+	-	-	-	IAA	BAP	MeJa+	+	+	+	-	-	Lght+
7	-	+	-	-	-	NAA	DHZ	MeJa-	+	-	-	+	+	Lght+
8	-	-	-	+	-	IAA	BAP	MeJa+	+	-	+	-	+	Lght-
9	+	+	+	+	+	2,4-D	DHZ	MeJa-	+	+	+	-	+	Lght+
10	+	-	+	+	+	NAA	DHZ	MeJa+	-	+	-	-	-	Lght-
11	-	-	+	+	+	NAA	DHZ	MeJa-	+	-	+	+	+	Lght-
12	-	+	+	-	-	NAA	DHZ	MeJa-	-	+	+	+	-	Lght-
13	-	-	-	+	+	IBA	Kinetin	MeJa-	-	-	-	+	+	Lght-
14	+	-	-	+	-	IBA	DHZ	MeJa-	-	+	+	+	-	Lght-
15	+	+	-	+	-	IBA	BAP	MeJa+	+	-	-	-	+	Lght+
16	-	-	-	-	-	2,4-D	DHZ	MeJa+	-	-	+	+	+	Lght-
17	+	-	-	-	+	IBA	Kinetin	MeJa+	-	+	+	+	+	Lght+
18	-	-	+	-	+	2,4-D	Kinetin	MeJa-	-	+	+	-	-	Lght-
19	-	+	+	-	-	IBA	DHZ	MeJa-	-	+	-	+	+	Lght+
20	+	+	+	+	+	IBA	Kinetin	MeJa+	+	+	-	+	-	Lght-
21	+	+	+	-	-	IBA	Kinetin	MeJa+	+	+	+	-	-	Lght-
22	+	-	+	-	+	IBA	Kinetin	MeJa-	+	+	-	-	+	Lght+
23	+	+	-	-	+	IBA	DHZ	MeJa-	+	-	+	+	+	Lght-
24	+	-	-	+	+	NAA	DHZ	MeJa+	+	-	+	-	-	Lght+
25	-	-	-	+	+	2,4-D	BAP	MeJa-	-	-	-	-	+	Lght+
26	+	-	+	-	-	NAA	Kinetin	MeJa+	-	-	+	-	+	Lght-

Run name	KNO_3	NH_4NO_3	$CaCl_2$	KH_2PO_4	$MgSO_4$	Auxins	Cytokinins	MeJa	SA	GA_3	Ethephon	Cyclanilide	ABA	Light
27	+	-	+	-	-	2,4-D	DHZ	MeJa-	-	-	+	-	-	Lght+
28	-	-	-	+	+	NAA	BAP	MeJa+	-	+	-	-	+	Lght-
29	+	-	+	-	+	IAA	DHZ	MeJa+	+	+	+	+	-	Lght-
30	+	+	-	+	+	2,4-D	BAP	MeJa+	-	+	+	+	+	Lght-
31	-	+	+	+	-	NAA	BAP	MeJa+	+	+	-	+	-	Lght+
32	+	+	+	-	+	IAA	BAP	MeJa-	-	-	-	-	+	Lght+
33	-	-	-	+	-	IBA	BAP	MeJa-	+	+	+	+	+	Lght-
34	-	+	-	+	+	2,4-D	DHZ	MeJa+	+	+	+	+	-	Lght-
35	+	+	+	+	-	IAA	DHZ	MeJa+	-	-	+	+	+	Lght-
36	-	+	-	+	+	NAA	Kinetin	MeJa-	+	+	-	-	+	Lght+
37	+	-	-	-	+	IAA	DHZ	MeJa+	-	-	-	+	+	Lght+
38	+	-	+	-	-	IAA	DHZ	MeJa-	-	-	-	-	-	Lght-
39	+	+	-	-	-	NAA	DHZ	MeJa+	-	+	+	-	+	Lght+
40	+	-	+	+	-	2,4-D	BAP	MeJa+	+	+	+	-	-	Lght+
41	-	+	-	+	-	IAA	DHZ	MeJa-	-	-	+	-	-	Lght+
42	+	-	+	-	+	2,4-D	DHZ	MeJa+	+	+	-	+	+	Lght+
43	+	+	+	+	-	IBA	BAP	MeJa+	-	-	+	+	-	Lght+
44	-	-	+	-	-	NAA	BAP	MeJa+	+	-	-	-	+	Lght+
45	+	-	-	+	-	NAA	DHZ	MeJa-	-	+	-	+	+	Lght+
46	-	+	+	-	+	IAA	Kinetin	MeJa-	+	-	+	-	+	Lght-
47	+	-	-	-	-	NAA	Kinetin	MeJa-	+	-	-	-	-	Lght+
48	-	-	+	-	+	IAA	BAP	MeJa+	-	+	+	+	+	Lght-
49	-	+	+	+	+	2,4-D	DHZ	MeJa+	-	-	-	+	+	Lght+
50	-	-	+	+	-	2,4-D	Kinetin	MeJa-	-	+	-	+	-	Lght-
51	-	+	+	+	+	IBA	BAP	MeJa-	-	+	+	-	-	Lght+
52	-	-	+	+	-	IBA	DHZ	MeJa+	-	+	+	-	+	Lght+
53	+	-	-	-	-	2,4-D	DHZ	MeJa-	+	+	-	-	-	Lght-
54	-	+	-	+	-	IBA	Kinetin	MeJa+	-	-	+	-	+	Lght-

Run name	KNO_3	NH_4NO_3	$CaCl_2$	KH_2PO_4	$MgSO_4$	Auxins	Cytokinins	MeJa	SA	GA_3	Ethephon	Cyclanilide	ABA	Light
55	-	+	-	+	-	2,4-D	DHZ	MeJa-	-	-	-	-	-	Lght-
56	-	-	+	+	+	2,4-D	BAP	MeJa-	+	-	+	+	-	Lght+
57	+	-	+	+	+	2,4-D	Kinetin	MeJa+	-	-	-	-	-	Lght+
58	+	-	-	+	+	2,4-D	Kinetin	MeJa-	+	-	+	-	+	Lght-
59	+	+	-	-	-	IAA	Kinetin	MeJa-	-	+	-	+	-	Lght-
60	-	+	+	-	+	NAA	DHZ	MeJa+	+	-	-	-	-	Lght-
61	-	-	+	-	-	IAA	Kinetin	MeJa+	+	-	-	+	+	Lght-
62	-	+	+	-	+	IBA	DHZ	MeJa+	+	-	+	-	-	Lght+
63	-	-	-	-	-	IBA	Kinetin	MeJa-	-	-	+	+	-	Lght+
64	+	-	+	+	+	IAA	BAP	MeJa-	-	+	-	+	-	Lght+
65	-	-	+	+	-	IAA	BAP	MeJa+	-	-	-	+	-	Lght-
66	+	+	+	+	+	NAA	BAP	MeJa-	+	-	-	+	-	Lght-
67	+	+	+	-	-	2,4-D	BAP	MeJa+	+	-	+	-	+	Lght-
68	+	+	-	-	-	2,4-D	BAP	MeJa+	-	-	-	+	-	Lght-
69	-	-	-	+	-	NAA	Kinetin	MeJa+	+	+	+	-	-	Lght-
70	+	-	-	+	+	IBA	DHZ	MeJa+	+	-	-	-	-	Lght-
71	+	+	+	-	-	NAA	BAP	MeJa-	+	+	+	+	+	Lght-
72	+	+	-	+	+	NAA	BAP	MeJa-	-	-	+	-	+	Lght-
73	+	+	+	+	-	NAA	Kinetin	MeJa-	-	-	+	+	-	Lght+
74	+	-	-	-	-	IBA	BAP	MeJa+	+	+	-	+	-	Lght+
75	+	+	+	-	+	NAA	Kinetin	MeJa-	-	-	-	+	+	Lght-
76	-	-	-	-	-	NAA	BAP	MeJa+	-	-	+	+	-	Lght+
77	-	-	-	-	+	IBA	BAP	MeJa-	+	-	-	+	-	Lght-
78	+	+	-	-	+	2,4-D	Kinetin	MeJa+	+	-	+	+	-	Lght+
79	+	+	-	-	+	IAA	BAP	MeJa-	+	-	+	+	-	Lght+
80	-	+	-	-	+	2,4-D	BAP	MeJa-	-	+	-	+	-	Lght+
81	+	-	+	+	-	IAA	Kinetin	MeJa-	+	+	+	+	+	Lght+
82	-	+	-	-	+	IBA	DHZ	MeJa+	-	+	-	-	-	Lght-

Run name	KNO_3	NH_4NO_3	$CaCl_2$	KH_2PO_4	$MgSO_4$	Auxins	Cytokinins	MeJa	SA	GA_3	Ethephon	Cyclanilide	ABA	Light
83	+	+	-	+	-	IAA	BAP	MeJa-	+	+	-	-	+	Lght-
84	+	+	-	+	+	IAA	Kinetin	MeJa-	-	+	+	-	-	Lght-
85	-	-	+	-	+	IBA	BAP	MeJa-	-	-	+	-	+	Lght-
86	-	+	+	-	-	2,4-D	Kinetin	MeJa+	-	+	-	-	+	Lght+
87	-	+	-	-	+	IAA	Kinetin	MeJa+	-	-	-	-	-	Lght+
88	-	-	+	-	-	2,4-D	BAP	MeJa-	+	+	-	-	+	Lght-
89	-	-	-	-	+	NAA	Kinetin	MeJa+	+	+	-	+	-	Lght-
90	+	+	-	+	-	2,4-D	Kinetin	MeJa+	+	-	-	+	+	Lght-
91	-	+	+	+	-	IAA	DHZ	MeJa-	+	+	-	-	-	Lght-
92	+	-	+	+	-	IBA	DHZ	MeJa-	+	-	-	+	+	Lght+
93	+	-	-	-	+	NAA	BAP	MeJa-	-	+	+	-	-	Lght+
94	+	+	+	-	+	IBA	BAP	MeJa+	-	+	-	-	+	Lght-
95	-	-	+	+	+	IAA	Kinetin	MeJa+	+	-	+	+	-	Lght+
96	-	+	+	+	-	IBA	Kinetin	MeJa-	+	-	-	-	-	Lght+

Figure S1. NH_4NO_3 S-plot

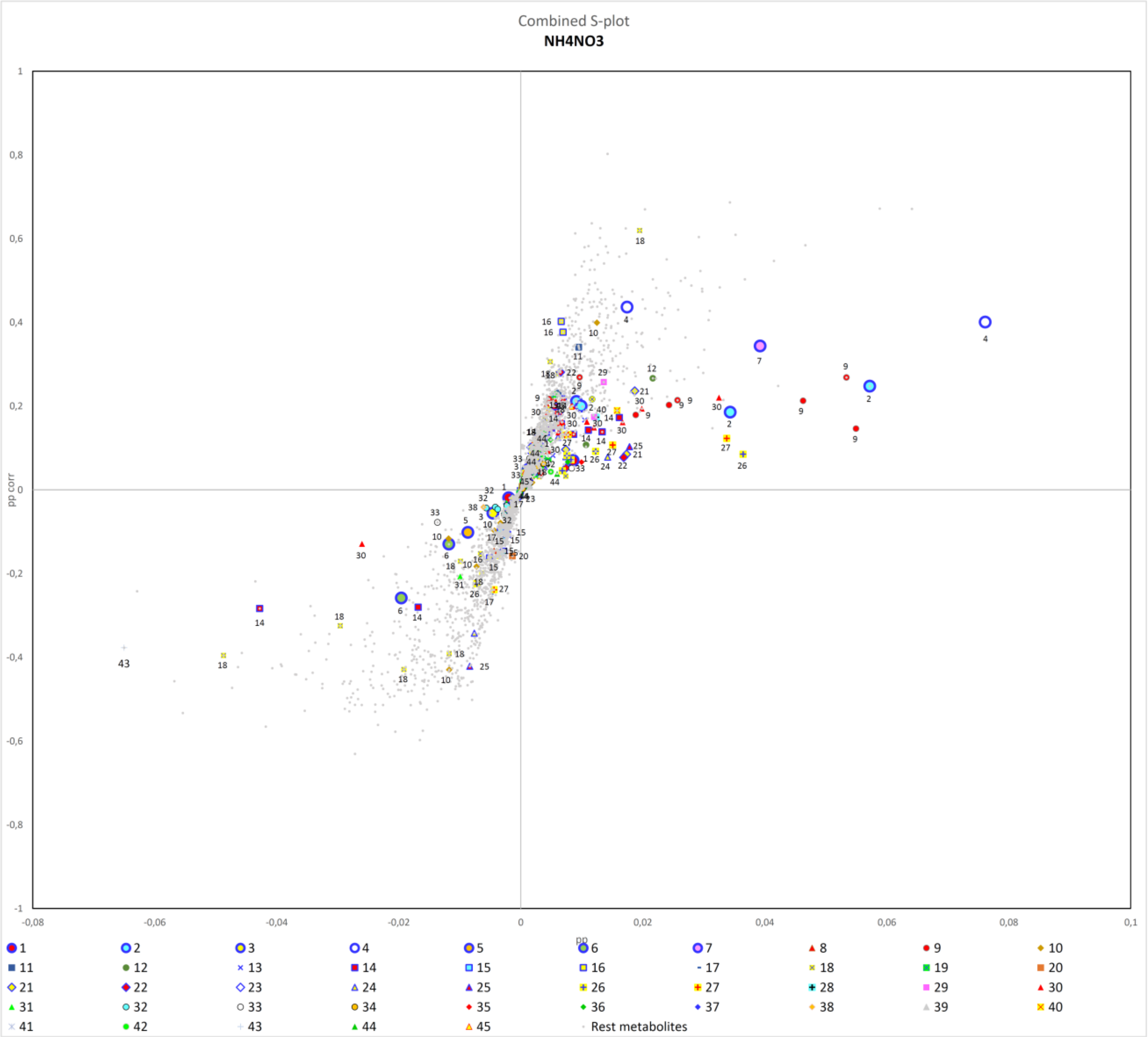

Figure S2. $CaCl_2$ S-plot

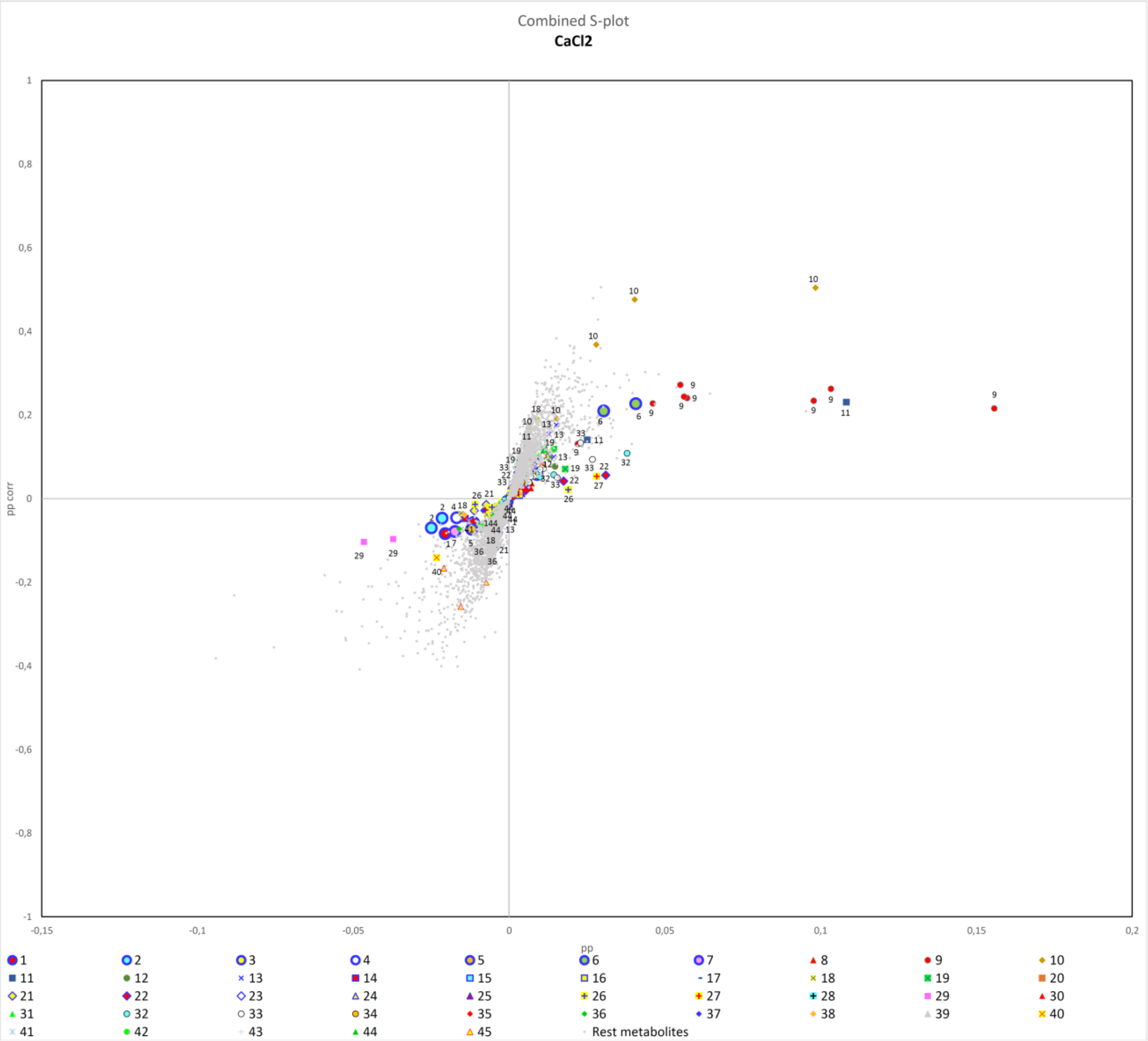

Figure S3. IAA S-plot

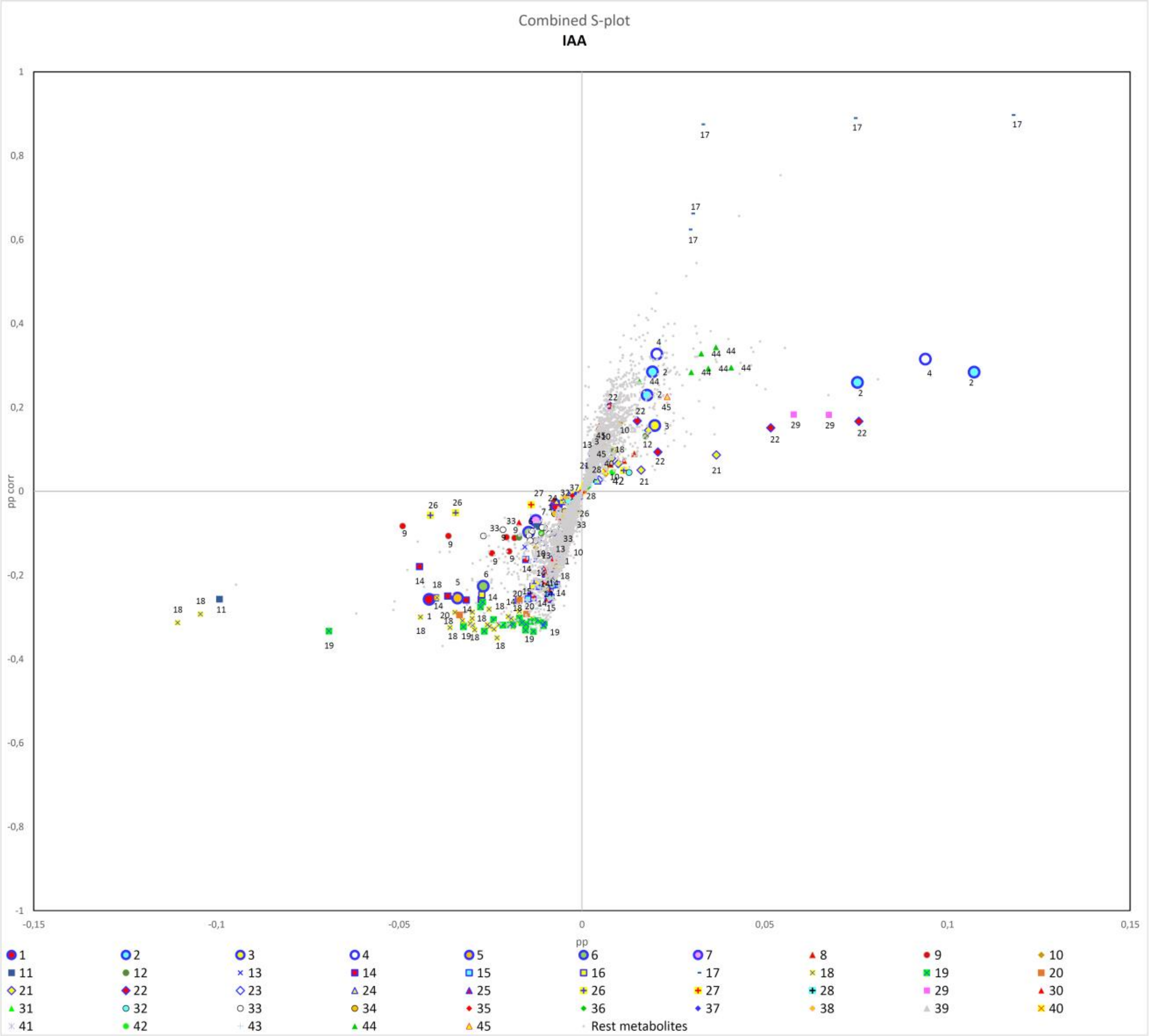

Figure S4. IBA S-plot

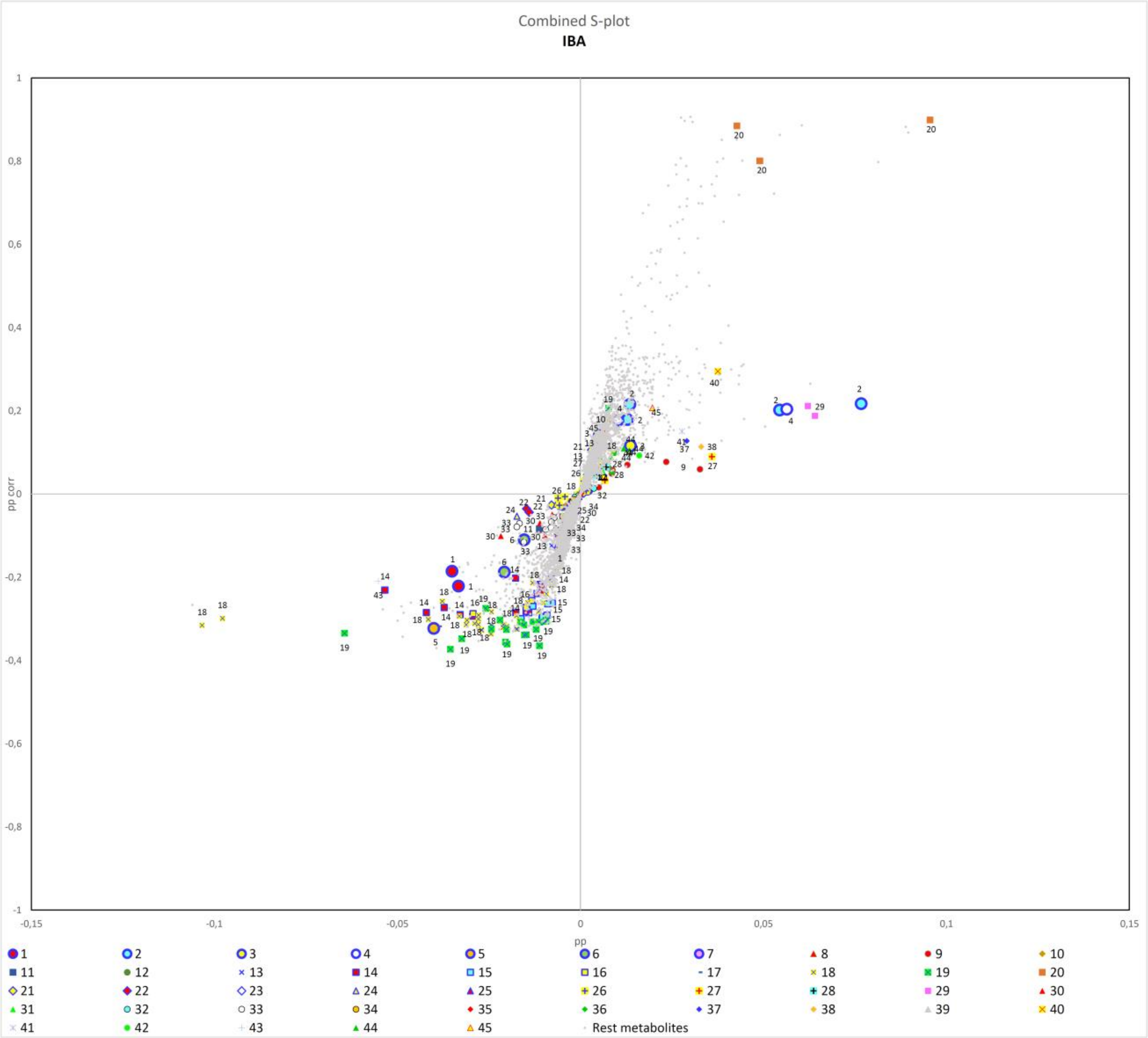

Figure S5. NAA S-plot

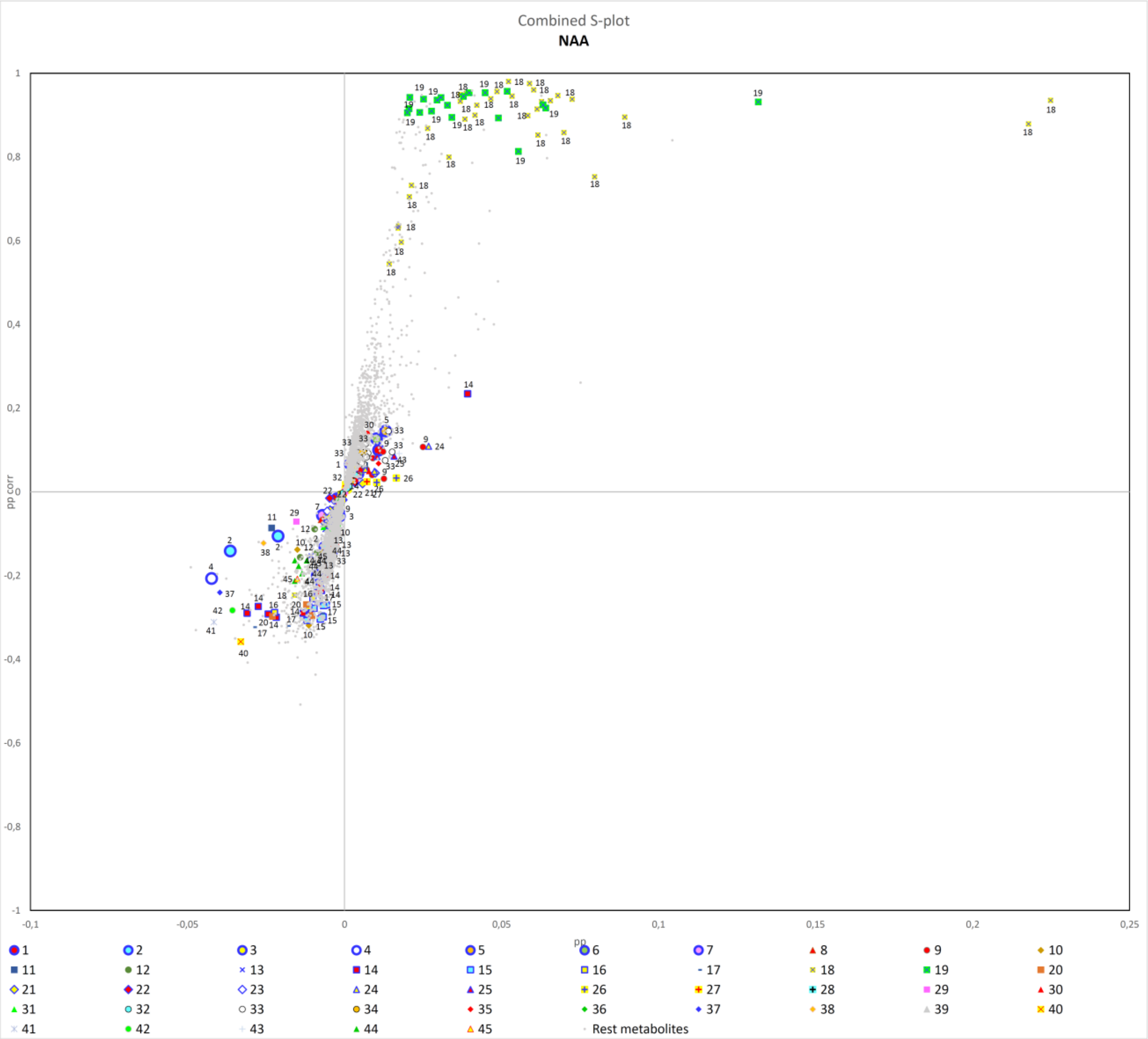

Figure S6. 2,4-D S-plot

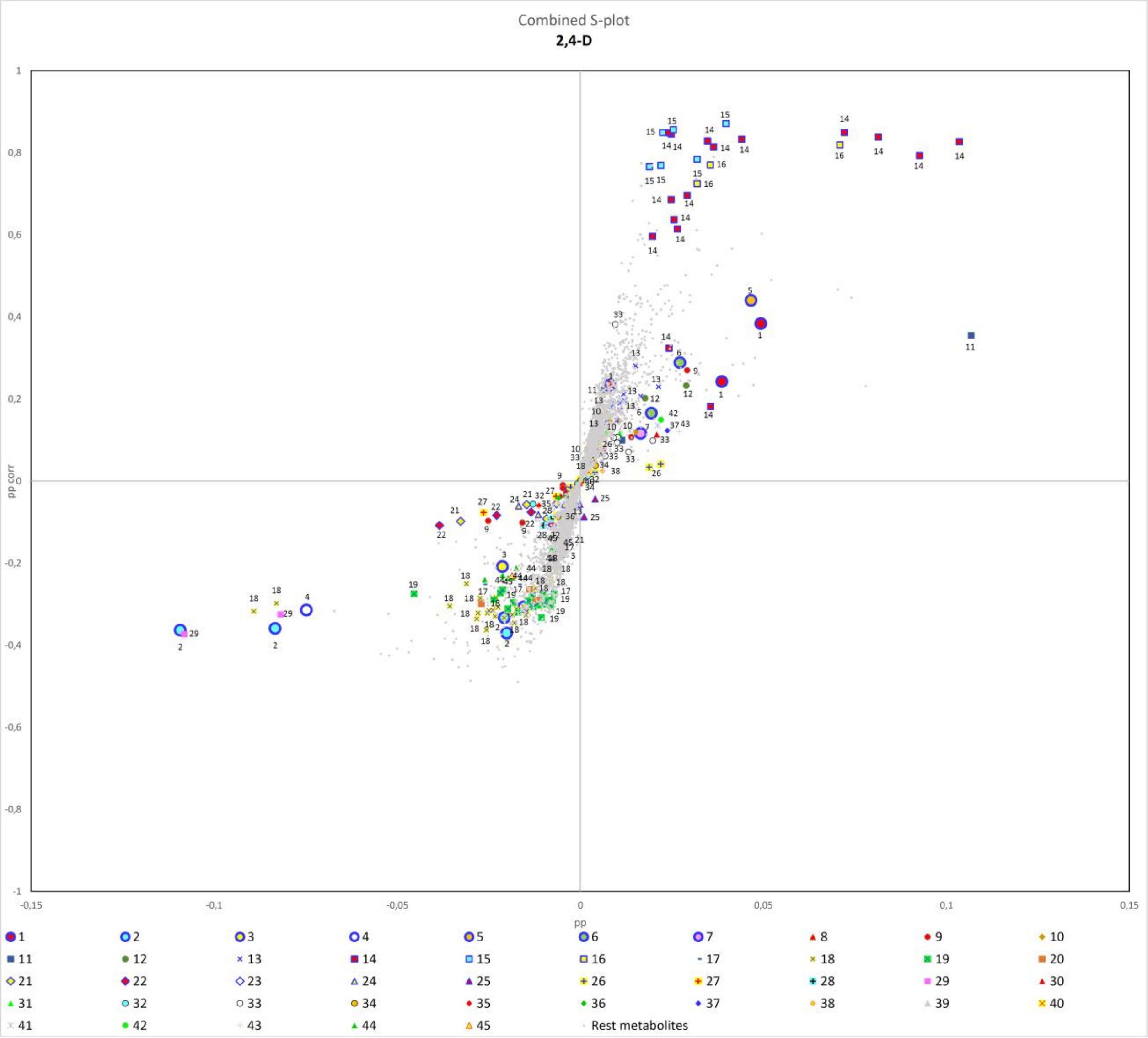

Figure S7. Kinetin S-plot

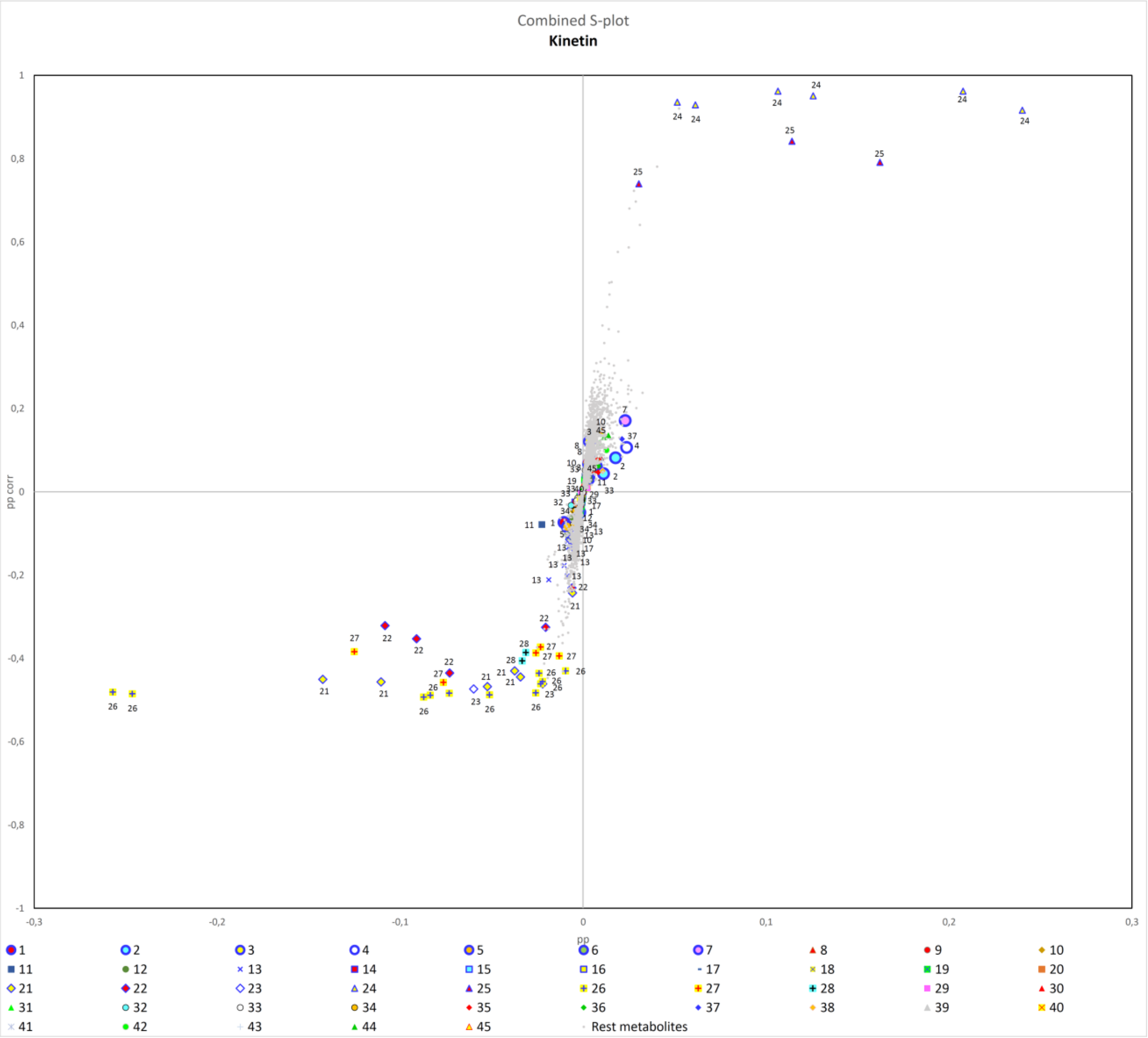

Figure S8. DHZ S-plot

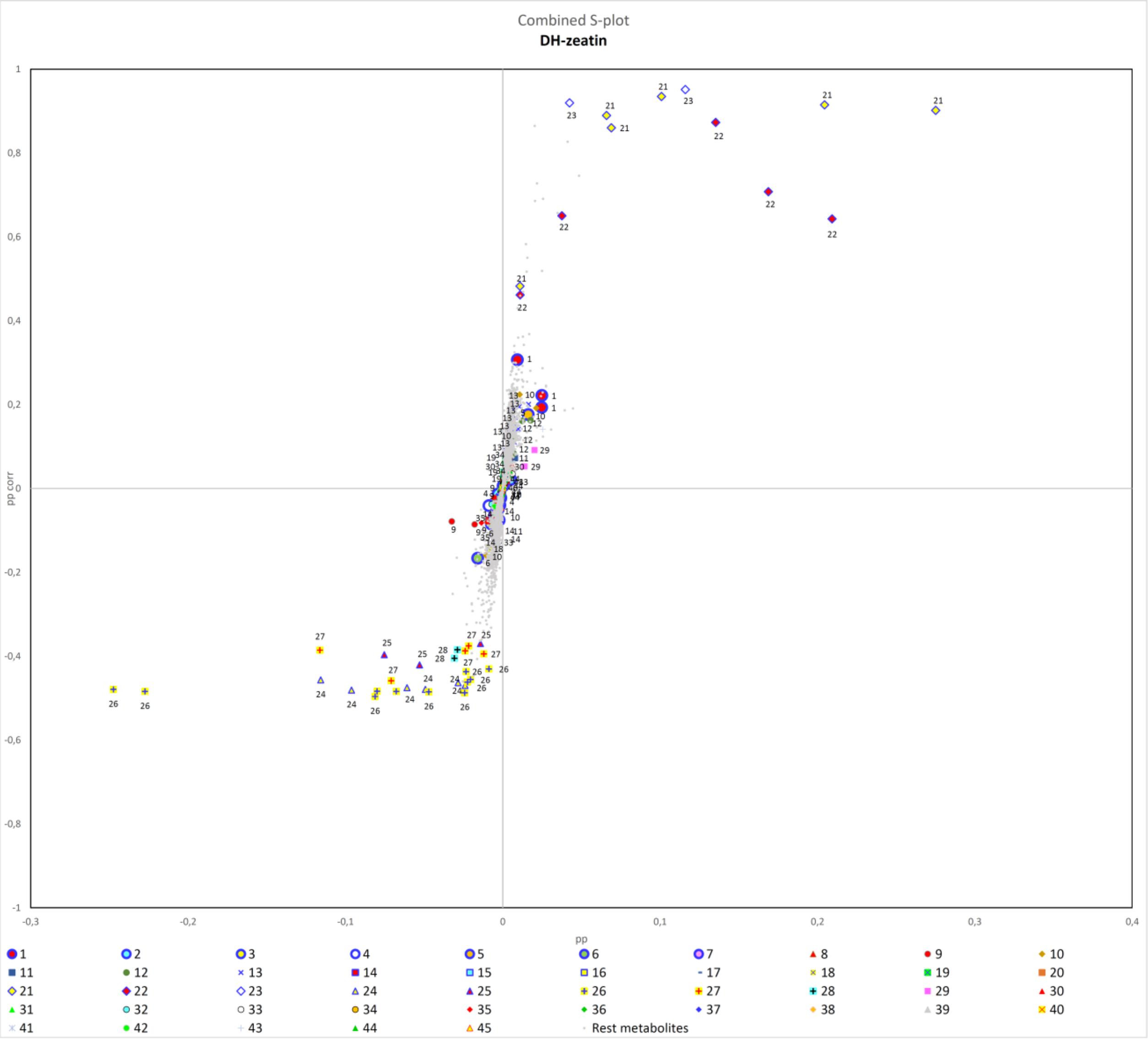

Figure S9. BAP S-plot

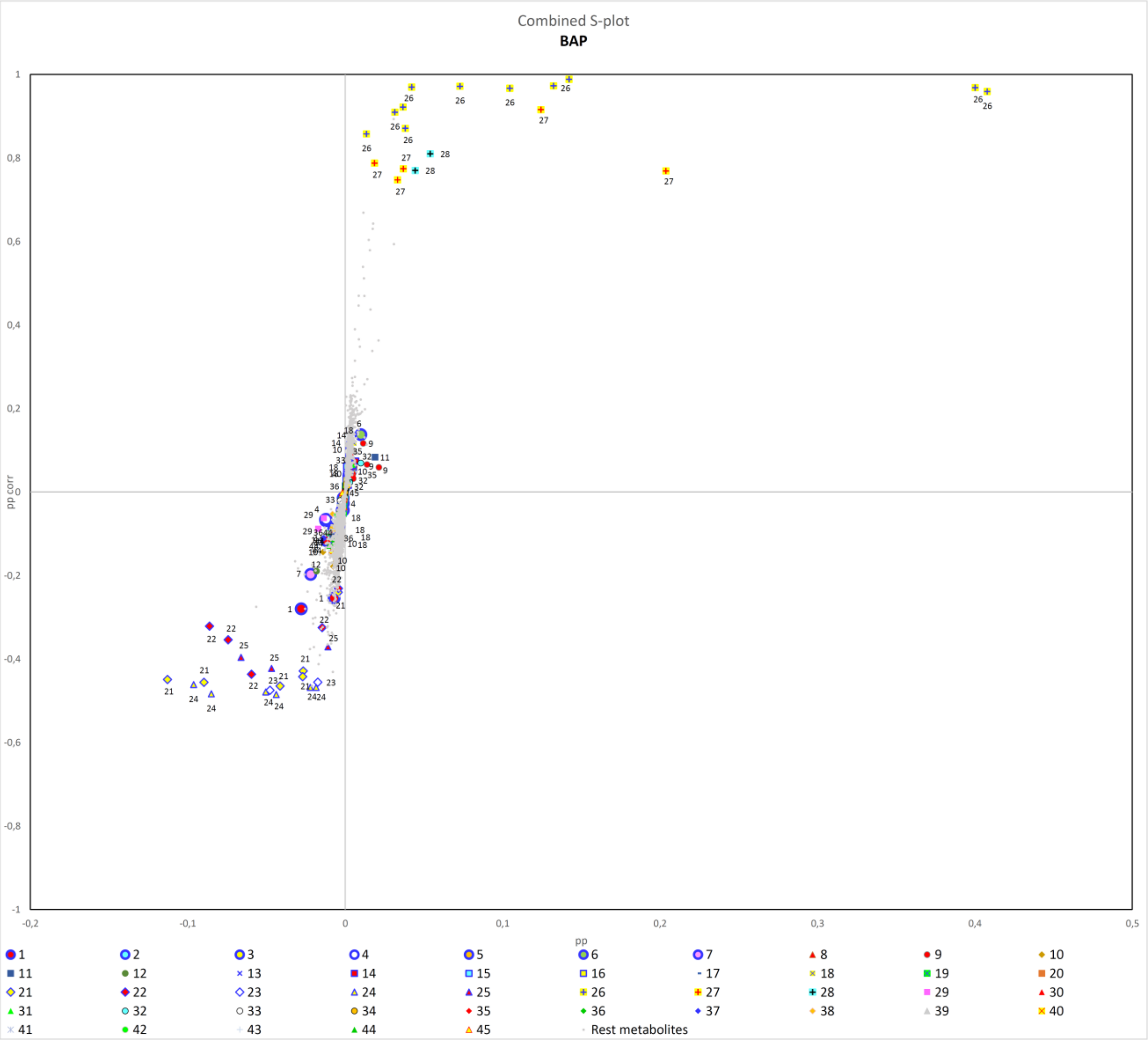

Figure S10. Methyl jasmonate S-plot

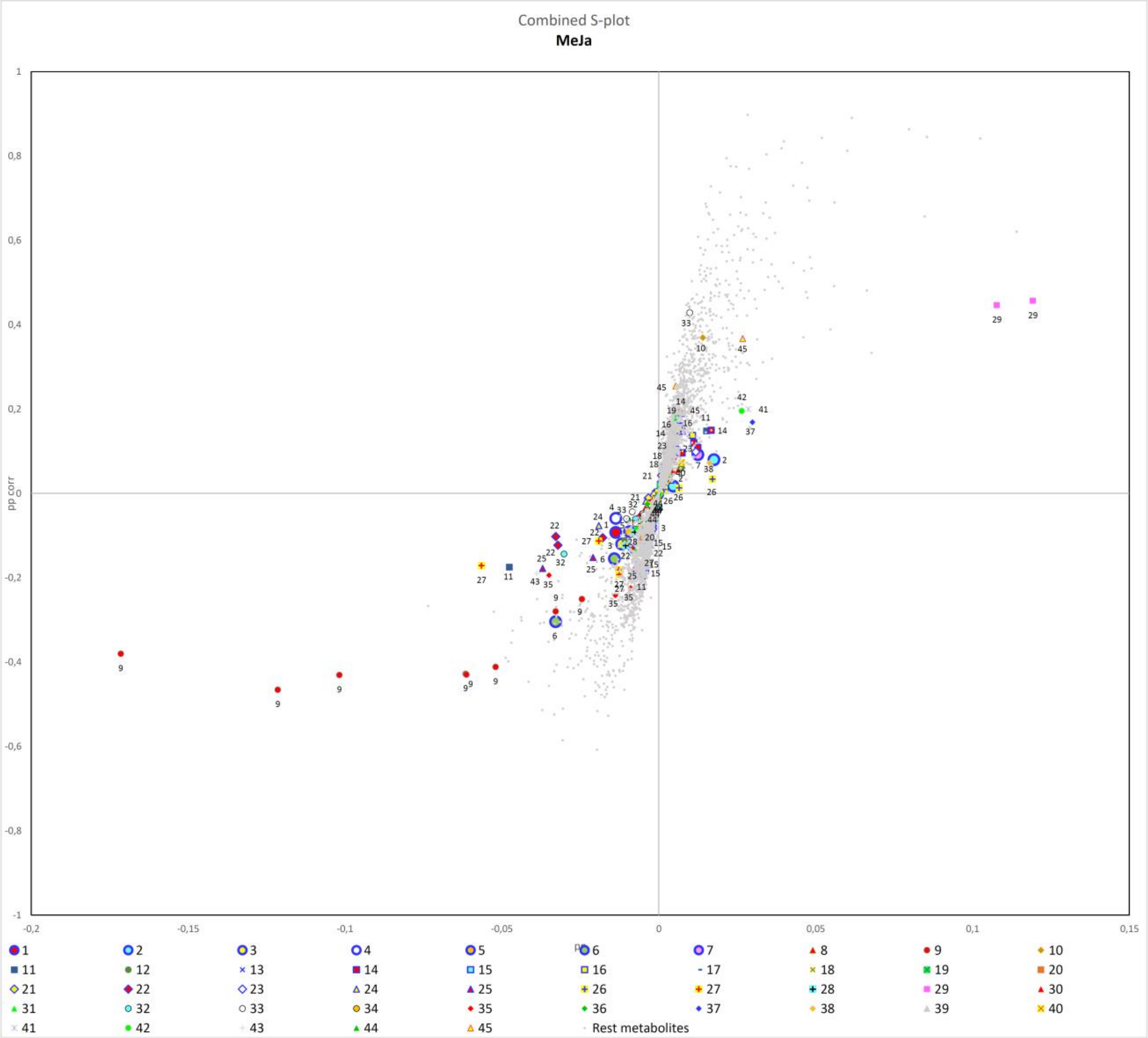

Figure S11. Salicylic acid S-plot

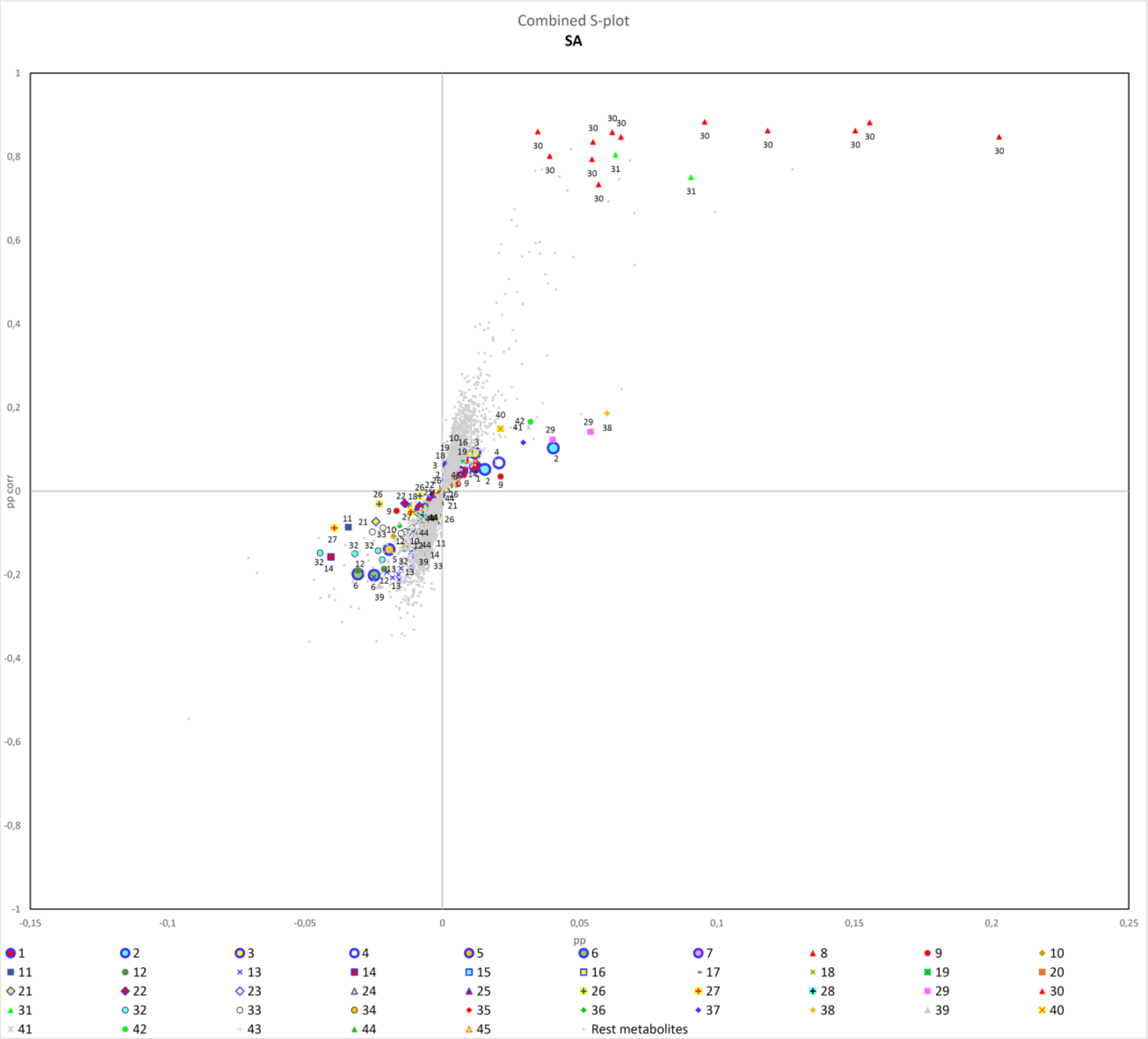

Figure S12. GA_3 S-plot

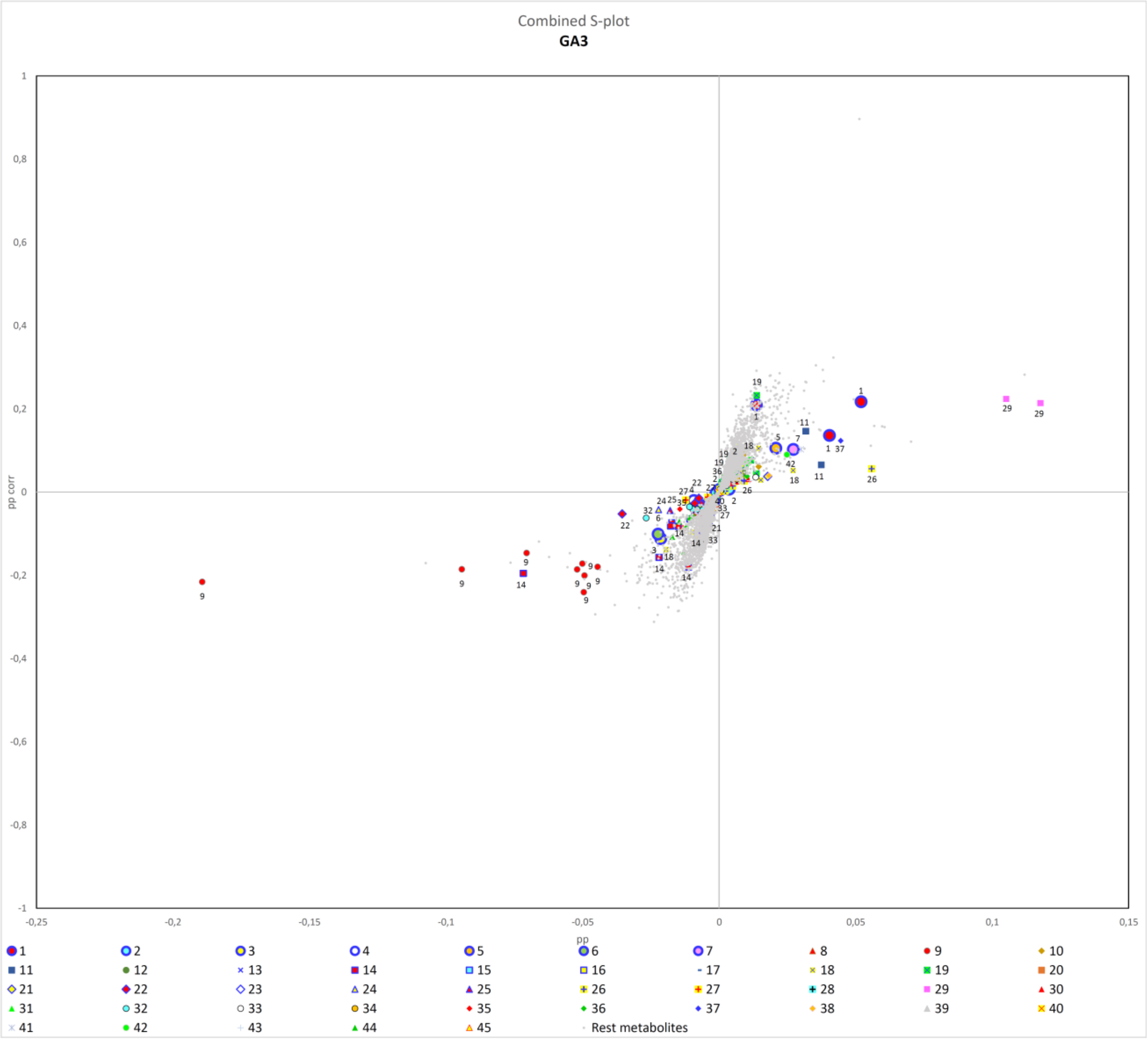

Figure S13. Ethephon S-plot

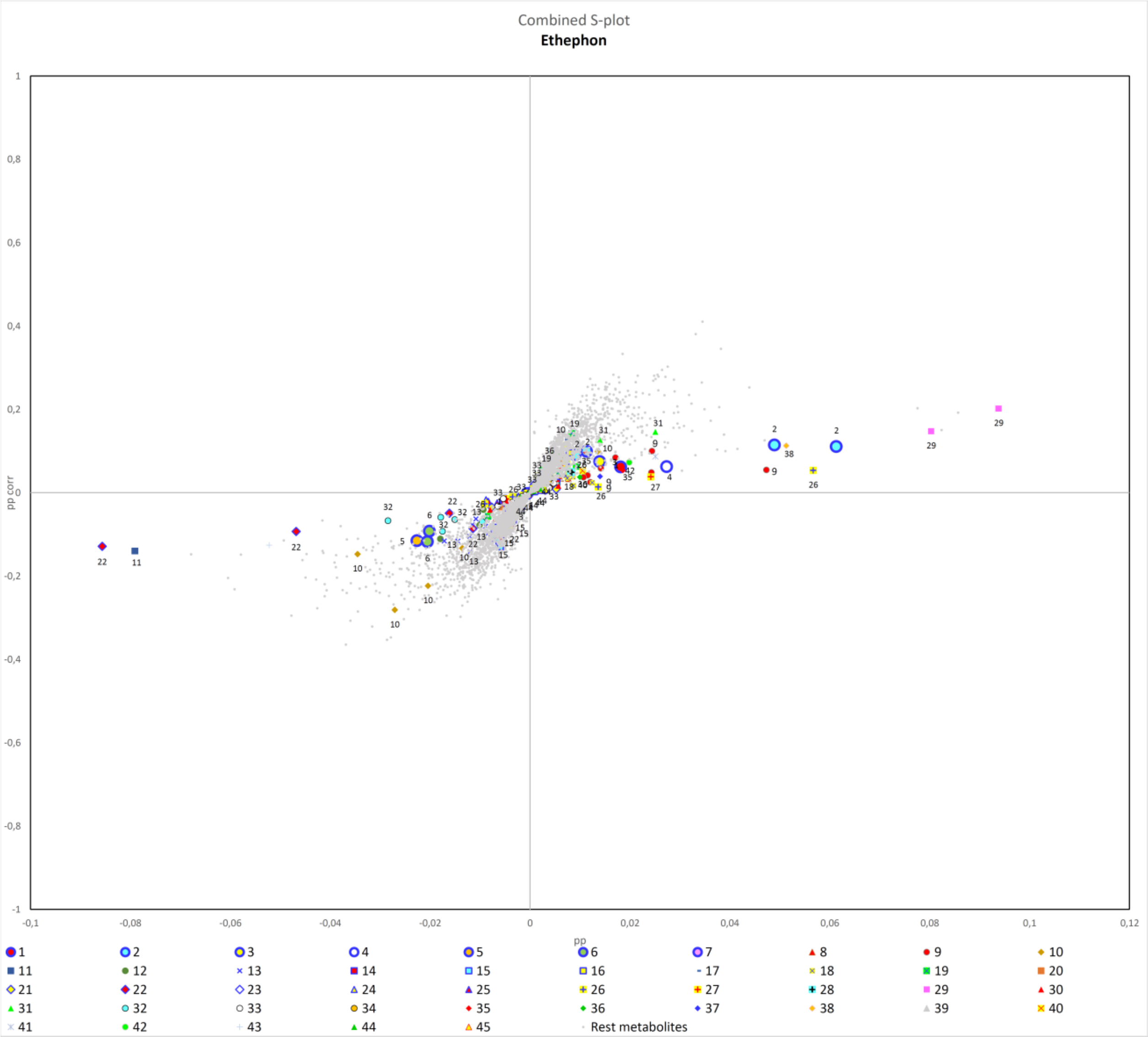

Figure S14. Cyclanilide S-plot

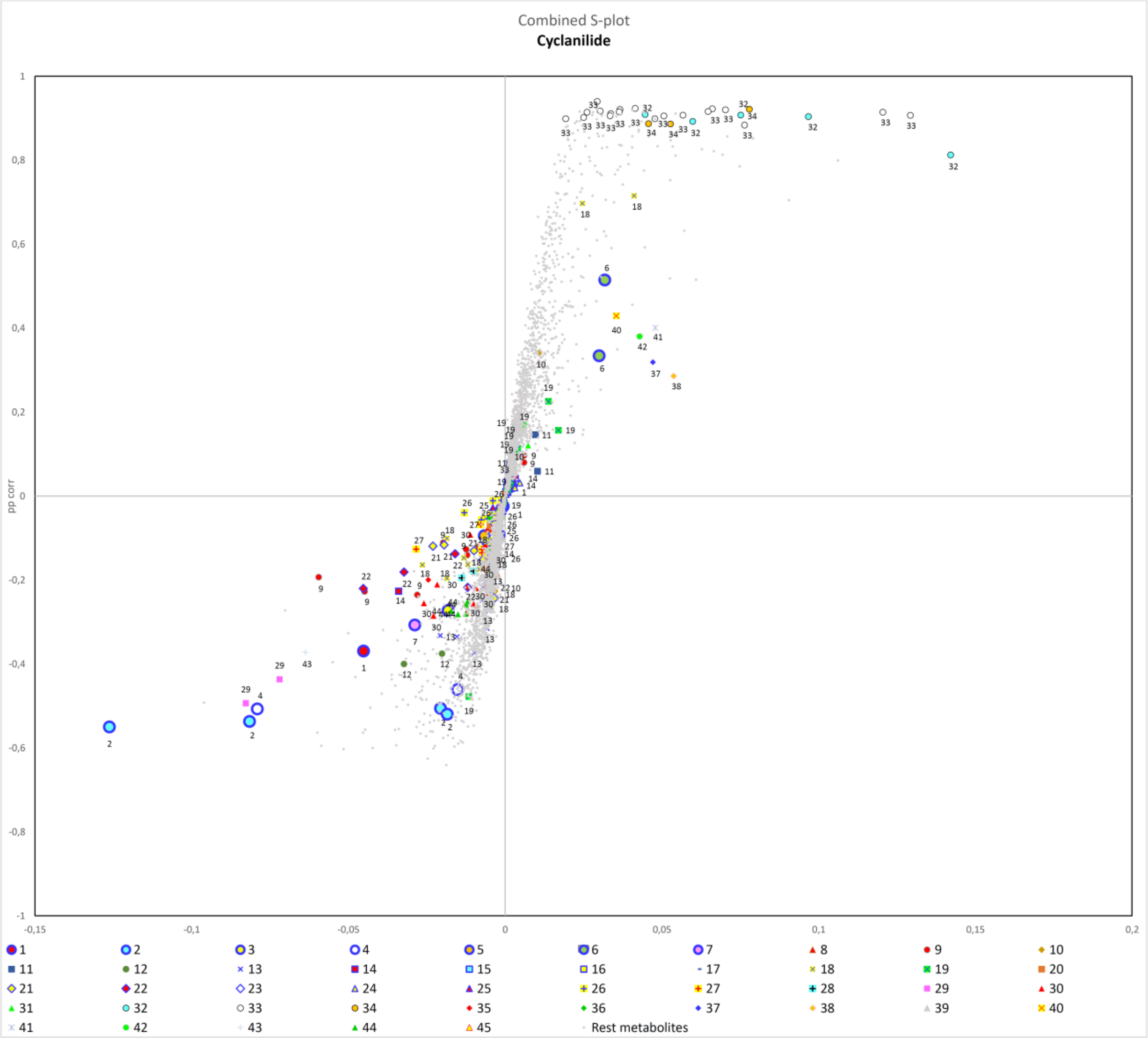

Figure S15. ABA S-plot

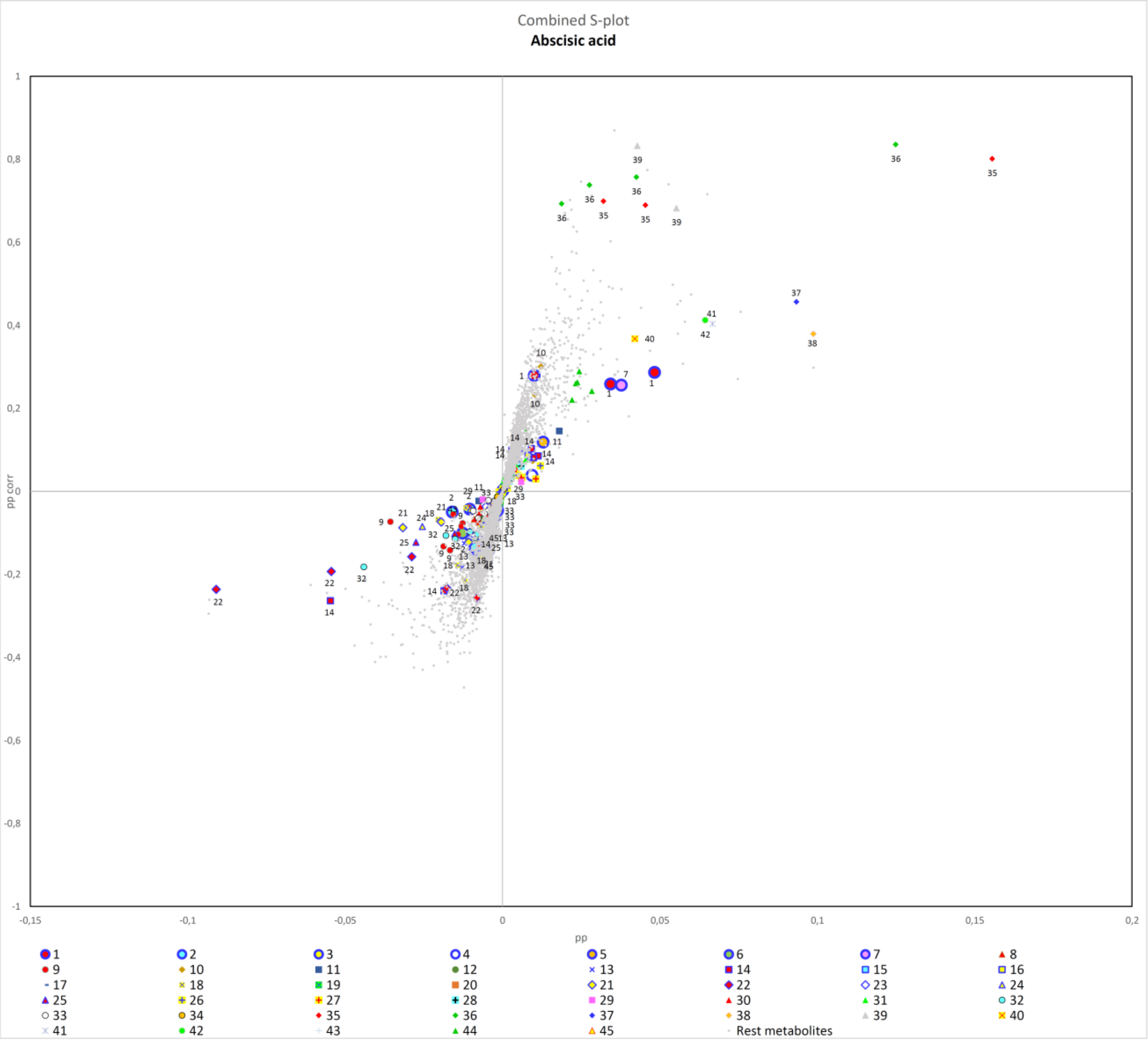

Figure S16. Light S-plot

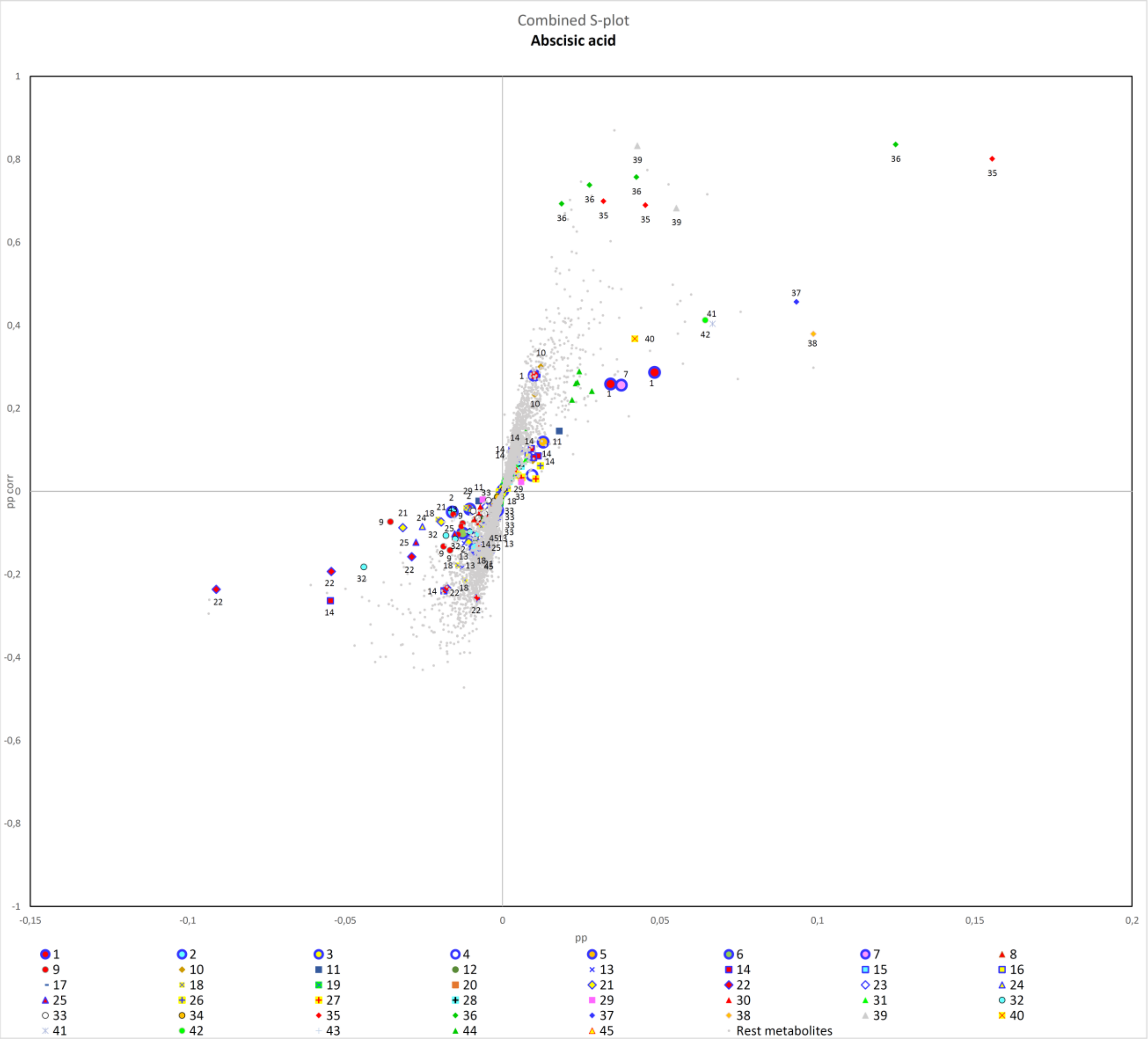

Figure S17. Plot of absolute correlations showing potentially relevant confounding between main effects and two-factor interactions in terms of triples of factor comparisons

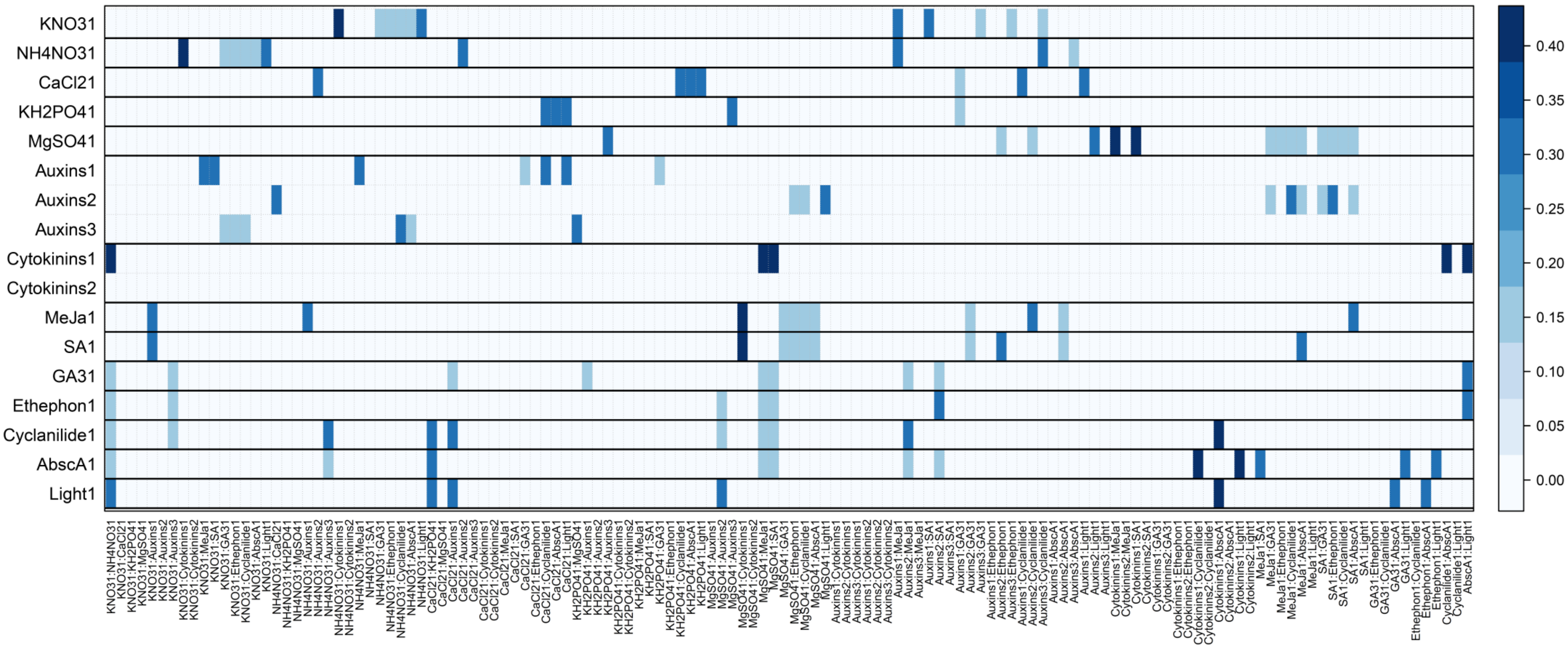

Supplement: Supplementary Information [file srep37390-s1.pdf]
